# Signature Patterns of MHC Diversity in Three Gombe Communities of Wild Chimpanzees Reflect Fitness in Reproduction and Immune Defense against SIVcpz

**DOI:** 10.1371/journal.pbio.1002144

**Published:** 2015-05-28

**Authors:** Emily E. Wroblewski, Paul J. Norman, Lisbeth A. Guethlein, Rebecca S. Rudicell, Miguel A. Ramirez, Yingying Li, Beatrice H. Hahn, Anne E. Pusey, Peter Parham

**Affiliations:** 1 Department of Structural Biology and Department of Microbiology and Immunology, Stanford University School of Medicine, Stanford, California, United States of America; 2 Vaccine Research Center, National Institutes of Health, Bethesda, Maryland, United States of America; 3 Sanofi, Cambridge, Massachusetts, United States of America; 4 Departments of Medicine and Microbiology, Perelman School of Medicine, University of Pennsylvania, Philadelphia, Pennsylvania, United States of America; 5 Department of Evolutionary Anthropology, Duke University, Durham, North Carolina, United States of America; Princeton University, UNITED STATES

## Abstract

Major histocompatibility complex (MHC) class I molecules determine immune responses to viral infections. These polymorphic cell-surface glycoproteins bind peptide antigens, forming ligands for cytotoxic T and natural killer cell receptors. Under pressure from rapidly evolving viruses, hominoid MHC class I molecules also evolve rapidly, becoming diverse and species-specific. Little is known of the impact of infectious disease epidemics on MHC class I variant distributions in human populations, a context in which the chimpanzee is the superior animal model. Population dynamics of the chimpanzees inhabiting Gombe National Park, Tanzania have been studied for over 50 years. This population is infected with SIVcpz, the precursor of human HIV-1. Because HLA-B is the most polymorphic human MHC class I molecule and correlates strongly with HIV-1 progression, we determined sequences for its ortholog, Patr-B, in 125 Gombe chimpanzees. Eleven Patr-B variants were defined, as were their frequencies in Gombe’s three communities, changes in frequency with time, and effect of SIVcpz infection. The growing populations of the northern and central communities, where SIVcpz is less prevalent, have stable distributions comprising a majority of low-frequency Patr-B variants and a few high-frequency variants. Driving the latter to high frequency has been the fecundity of immigrants to the northern community, whereas in the central community, it has been the fecundity of socially dominant individuals. In the declining population of the southern community, where greater SIVcpz prevalence is associated with mortality and emigration, Patr-B variant distributions have been changing. Enriched in this community are Patr-B variants that engage with natural killer cell receptors. Elevated among SIVcpz-infected chimpanzees, the Patr-B*06:03 variant has striking structural and functional similarities to HLA-B*57, the human allotype most strongly associated with delayed HIV-1 progression. Like HLA-B*57, Patr-B*06:03 correlates with reduced viral load, as assessed by detection of SIVcpz RNA in feces.

## Abstract 2

Wild chimpanzee populations maintain a diversity of major histocompatibility class I variants; one variant, enriched among chimpanzees infected with simian immunodeficiency virus, resembles the human variant that best impedes progression of HIV-1 infection.

## Author Summary

Polymorphic major histocompatibility complex (MHC) class I molecules activate immune responses against infection and correlate with susceptibilities to disease. In humans, longitudinal study of how disease epidemics alter MHC frequencies has not been possible. We studied chimpanzees, a species having direct equivalents of all human MHC class I genes. The wild Gombe chimpanzees are naturally infected with simian immunodeficiency virus (SIVcpz) and have been studied long-term. From samples of fecal DNA we sequenced *Patr-B*—the most polymorphic MHC gene—from 125 chimpanzees and identified eleven *Patr-B* alleles. Over a 15-year period, two of three social communities flourished, maintaining one or two high-frequency *Patr-B* alleles and many low-frequency alleles. The high frequencies were caused by the reproductive success of immigrants in one community and socially dominant, fecund individuals in the other. The third community declined, partly because of SIVcpz, experiencing greater change in *Patr-B* allele frequencies. In SIVcpz-infected chimpanzees, three *Patr-B* alleles are overrepresented, and one is underrepresented. Allele Patr-B*06:03 resembles HLA-B*57:01—the human MHC molecule that strongly resists HIV by reducing viral load. Patr-B*06:03 correlates with reduced SIVcpz load and likely lessens the impact of SIVcpz infection. HLA-B*57:01 and Patr-B*06:03 are related in structure, function and evolution, forming part of an exceptional trans-species group of hominid MHC-B alleles.

## Introduction

In vertebrate genomes, the major histocompatibility complex (MHC) is a region enriched with genes of the immune system. Defining the unique character of the MHC is the extreme polymorphism of the genes encoding the classical MHC class I and II molecules [1]. These cell-surface glycoproteins bind pathogen-derived peptide antigens and present them to the antigen receptors of T cells, the lymphocyte subpopulation that makes vital contributions to every arm of the adaptive immune response. The MHC class I molecules present peptide antigens to cytotoxic CD8 T cells, which can then kill cells infected with viruses and other types of intracellular pathogens [2]. In a complementary fashion, the peptide antigens bound by the MHC class II molecules stimulate CD4 T cells that then activate macrophages and B cells to respond to extracellular pathogens [3,4]. The activated B cells make antibodies, which coat the pathogen surface, thereby facilitating phagocytosis and pathogen destruction by an activated macrophage. The functions of MHC class II molecules are limited to adaptive immunity, whereas MHC class I molecules also make seminal contributions to innate immunity. Natural killer (NK) cells are the major blood lymphocytes of innate immunity; they recognize virus-infected cells and kill them by using various receptors that recognize MHC class I [5]. An advantage to this innate defense is its potential to terminate primary viral infections at a much earlier stage than adaptive immunity. Also, in placental mammals, NK cells and their receptors for MHC class I play a critical role in reproduction, specifically in the formation of the placenta [6].

Across phylogeny, the MHC class I genes are less conserved than MHC class II, both in their number and their nature [7]. This is consistent with MHC class I evolving faster than class II because of pressure from rapidly evolving intracellular pathogens such as RNA viruses, like the human immunodeficiency viruses (HIV). In fact, the great apes (chimpanzee, bonobo, gorilla, and orangutan) are the only living species that have orthologs of all three polymorphic human MHC class I molecules, HLA-A, -B, and -C [8]. In coevolving with MHC class I molecules, the Killer cell Immunoglobulin-like Receptor (KIR) family of NK cell receptors also evolves rapidly [6,9]. Only simian primates have counterparts to the human KIR family, and even within them there is remarkable species-specific character. Among the great apes, the immunogenetics, genomics, and functional interactions of chimpanzee KIR and MHC class I are most similar to the human system [8,10]. Thus the chimpanzee, *Pan troglodytes*, represents the best animal model in which to study the complex immunogenetics of MHC class I molecules and their diverse interactions with lymphocyte receptors.

The similarity of human and chimpanzee immune systems is reflected in the many pathogens the two species share, which in turn facilitates epidemiological studies to correlate host immunogenetic factors with infection. This is exemplified by pandemic (group M) HIV-1, which is derived from a simian immunodeficiency virus from chimpanzees (SIVcpz) that entered the human population by zoonotic transmission and causes acquired immunodeficiency syndrome (AIDS) [11]. Analysis of viral phylogeny shows that humans acquired ape viruses on four independent occasions, twice from the central subspecies of chimpanzee, *P. t. troglodytes*, generating HIV-1 groups M and N [11–13], and twice from western lowland gorillas (*Gorilla gorilla gorilla*), generating HIV-1 groups O and P [13–15]. However, no such transmissions occurred from the eastern subspecies of chimpanzees, *P. t. schweinfurthii*, which harbors SIVcpz at high prevalence [11,13,16]. Finally, the western *P. t. verus* and Nigeria-Cameroonian *P. t. ellioti* subspecies of chimpanzees are free of SIVcpz infection [13], which is an important distinction because most captive chimpanzees are of the *P. t. verus* subspecies [17,18] and thus have not experienced SIVcpz infection in their evolutionary history.

**Fig 1:**
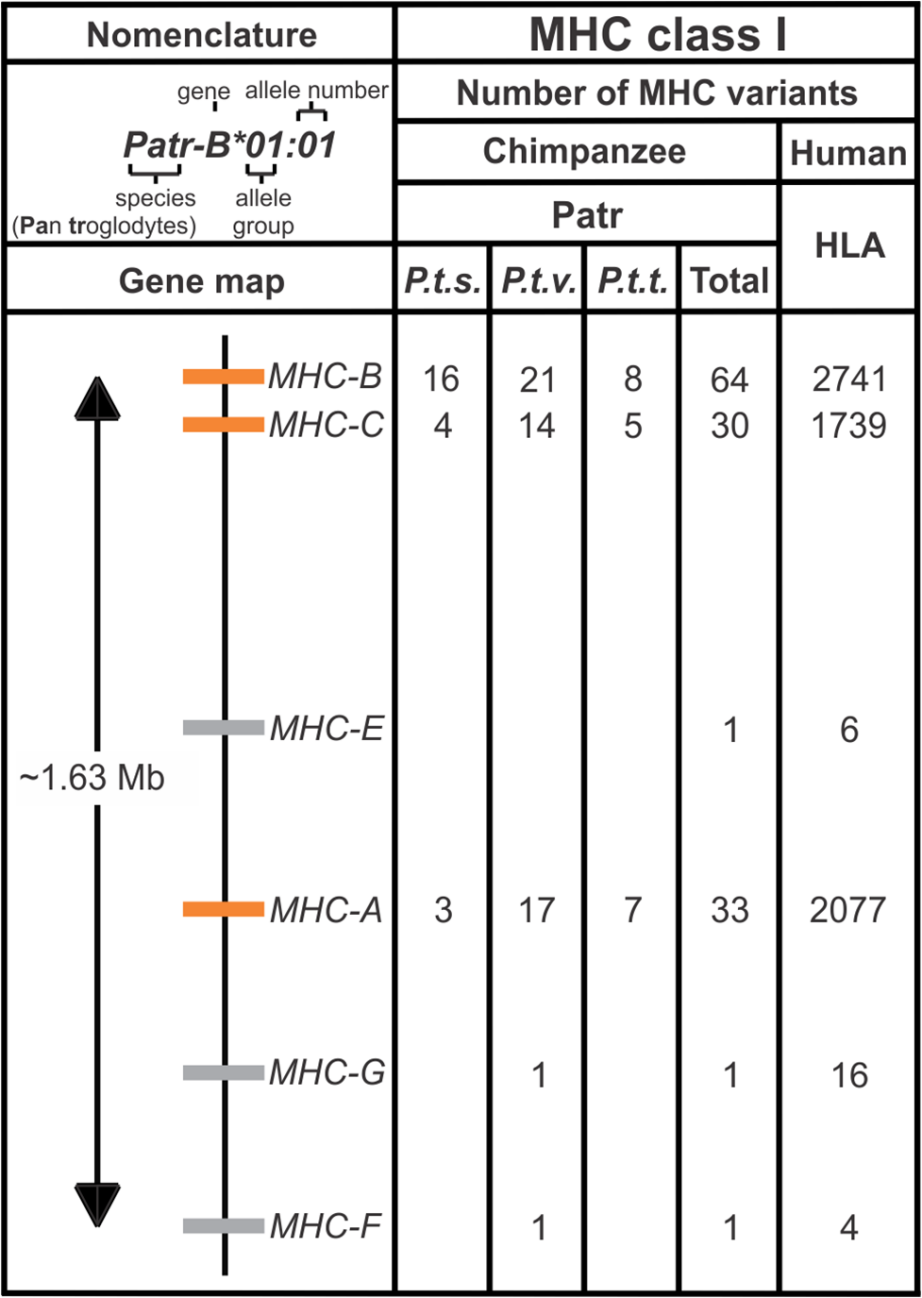
Organization and polymorphism of human and chimpanzee major histocompatibility complex (MHC) class I genes. Humans and chimpanzees have six orthologous MHC class I genes: *MHC-A, -B, -C, -E, -F,* and *-G*. Human genes and alleles have the prefix *HLA-* (Human Leukocyte Antigen). Chimpanzee genes and alleles have the prefix *Patr-* (*Pan troglodytes*). The gene map shows their relative organization within the MHC. Each gene encodes a cell-surface glycoprotein that is involved in activating cytotoxic T and NK cell responses against infection. *MHC-A, -B,* and -*C* (orange) are highly polymorphic; *MHC-E, F,* and *G* (gray) are conserved. Under “MHC class I” are given the number of protein variants (allotypes) so far defined for each gene. For chimpanzees, these numbers are given for the three subspecies studied: *P. t. schweinfurthii (P.t.s.)* (the number given includes the novel *Patr-B* alleles defined in this study), *P. t. verus (P.t.v.)*, and *P. t. troglodytes (P.t.t.)*. For the subspecies *P. t. ellioti*, MHC class I genes have yet to be studied. The sum of all chimpanzee allotypes identified is given under “Total.” The subspecies origin of the *Patr-E* variant sequenced is not known. Under “Nomenclature” is shown the standardized nomenclature for *Patr-B* alleles, the subject of this paper. Further information is given on the http://www.ebi.ac.uk/ipd/imgt/hla/ and http://www.ebi.ac.uk/ipd/mhc/ websites [21].

NK cells and cytotoxic T cells provide critical defense mechanisms against viral infections. Consistent with NK cell and T cell receptor recognition of antigens presented by MHC class I, the strongest host genetic correlations for HIV-1 progression are with *HLA-B* [19], the most polymorphic human *MHC class I* gene [8,20,21] (Fig 1). Study of MHC diversity in wild populations of apes and other primates has been limited to MHC class II because its polymorphism is less complicated and more readily determined [22–32]. Knowledge of Patr-B, the chimpanzee ortholog of HLA-B, has come from the study of B cell lines derived from the peripheral blood of captive chimpanzees, most of whose provenance was poorly documented. We, therefore, know little of Patr-B polymorphism in natural chimpanzee populations, how it compares to HLA-B polymorphism, or how it is affected by viral infection.

For investigating natural *Patr-B* polymorphism, the chimpanzee population of Gombe National Park, Tanzania, has several advantages. Since Jane Goodall initiated investigation of the Gombe chimpanzees more than 50 years ago [33], the composition of this population and its temporal dynamics have been well-characterized [34]. In the last 20 years, it became possible to study the genetics of the chimpanzees and their pathogens in a noninvasive way by using fecal droppings as a source of DNA and RNA [16,35–40]. This allowed the study of the natural history of SIVcpz infection in the Gombe population [16,37–39]. With this approach we have now determined the diversity, polymorphism, and population dynamics of *Patr-B* in the Gombe chimpanzees.

## Results

**Fig 2:**
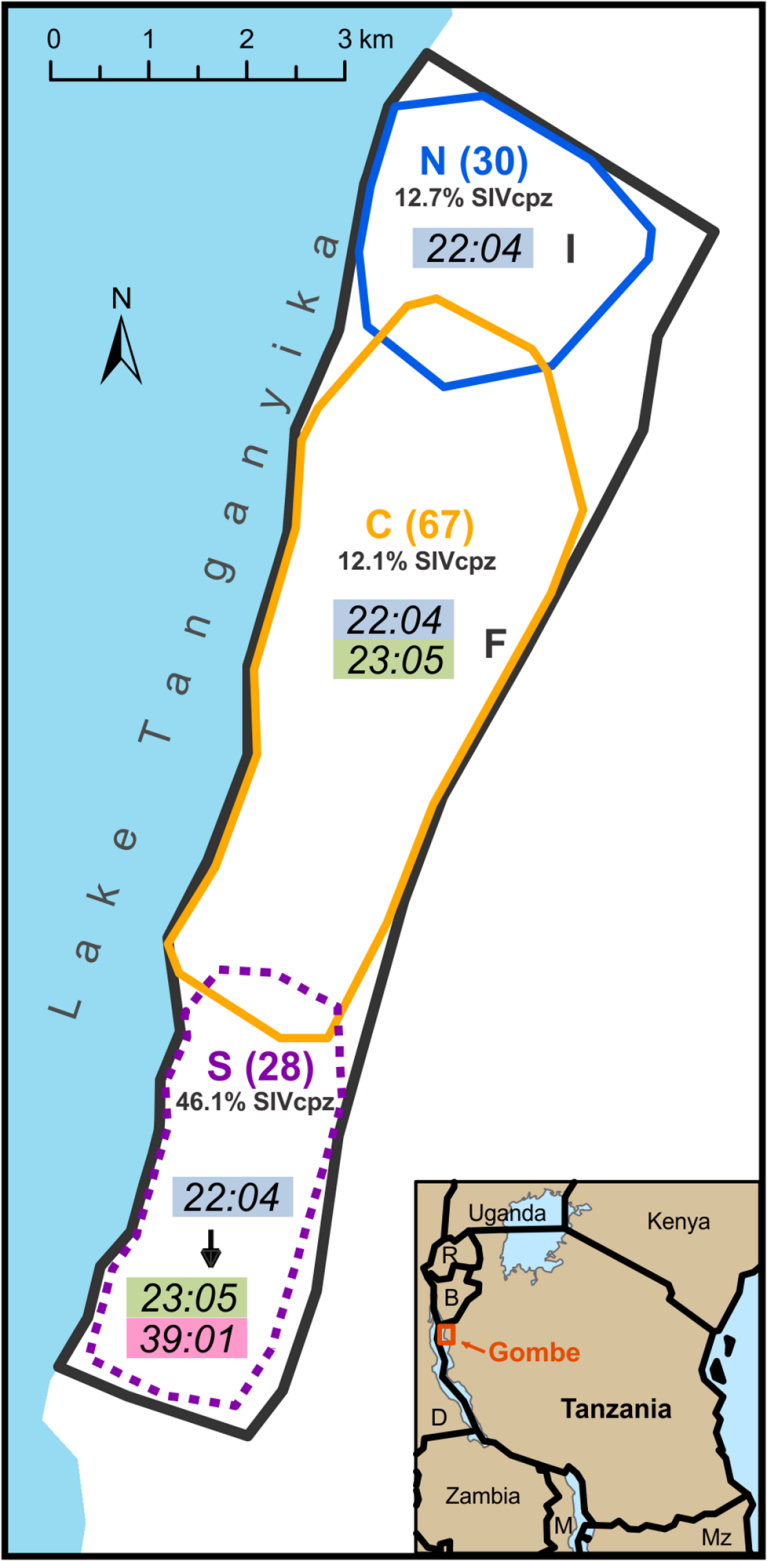
Territories of the three chimpanzee communities in Gombe National Park. Gombe National Park is small, covering 35 square kilometers of mountainous terrain in northwest Tanzania and has many streams that flow into Lake Tanganyika. The thick black line in the map shows the park boundary. The blue line denotes the territory in 2010 of the northern community (N), the orange line denotes the territory of the central community (C), and the dashed purple line denotes the approximate territory of the southern community (S). The inset map shows the location of the park, both within Tanzania and relative to other East African countries: Rwanda (R), Burundi (B), Democratic Republic of Congo (D), Malawi (M), and Mozambique (MZ). Within each community, the number of individuals sampled is given in parentheses, followed by the mean SIVcpz prevalence [16]. Shown for each community are the high-frequency *Patr-B* alleles. The expanding northern and central communities maintained the same high-frequency alleles throughout the study (1995–2010), while it changed in the southern community, as shown by the arrow. Immigrant females (indicated by “I”) and their progeny were responsible for the high-frequency allele in the north, whereas in the central community, that role was played by the progeny of fecund males and females (indicated by “F”). The map was created by Steffen Foerster, The Jane Goodall Institute Research Center, Duke University.

We investigated *MHC* polymorphism in the wild chimpanzee population of Gombe National Park in northwestern Tanzania. This population, of subspecies *P. t. schweinfurthii*, is divided into three social communities that live in largely separate, but overlapping, northern, central, and southern territories within the park (Fig 2). Of 125 individuals studied, 30 were first sampled in Mitumba (northern), 67 in Kasekela (central), and 28 in Kalande (southern). This first study of wild chimpanzee *MHC* focuses on the *Patr-B* gene because previous analyses of captive chimpanzees, mainly subspecies *P. t. verus*, showed that *Patr-B* is the most polymorphic chimpanzee *MHC* gene, and its human ortholog, *HLA-B*, is the most polymorphic gene in the human genome [8,20,21]. Polymorphism of MHC molecules is the property of the binding site domains, which determine the most functional variation. Exons 2 and 3 of *Patr-B*, which encode its α_1_ and α_2_ peptide-binding domains, respectively, were amplified, cloned, and sequenced from genomic DNA isolated from chimpanzee feces.

### The Gombe Chimpanzees Have Extensive Patr-B Diversity and Two High-Frequency Alleles

**Fig 3:**
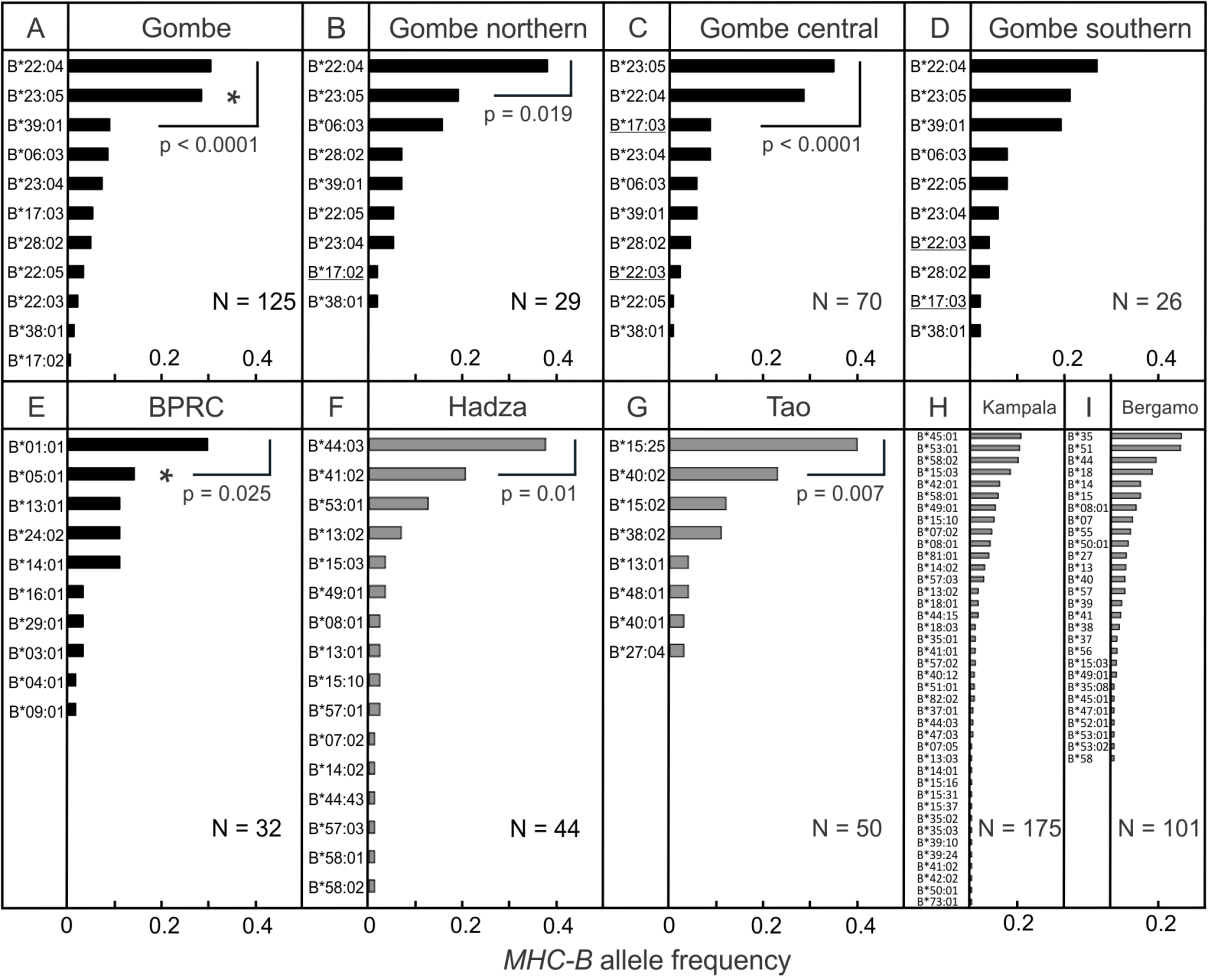
Frequency distribution of *MHC-B* alleles in chimpanzee and human populations. The upper panels give the *Patr-B* allele frequencies for the entire Gombe population (A), the northern community (B), the central community (C), and the southern community (D). The Gombe chimpanzees are of subspecies *P. t. schweinfurthii*. (B-D) Underlined are the names of alleles not present in all three communities. The lower panels show the *Patr-B* allele frequencies for the Biomedical Primate Research Centre (BPRC) chimpanzee population, subspecies *P. t. verus* [41] (E) (further described in S1 Text), and the *HLA-B* allele frequencies (in gray) in the Hadza [42] (F), Tao [43] (G), Kampala [43,44] (H), and Bergamo [43] (I) human populations. The Hadza and Tao are indigenous, tribal populations from Africa (Tanzania) and Asia (Taiwan), respectively; Kampala and Bergamo are admixed urban populations from Africa (Kampala, Uganda) and Europe (Bergamo, Italy), respectively. The wider brackets (A and C) show that the two most common alleles are significantly more frequent than all other alleles, but do not differ significantly from each other; the narrower brackets (B, E, F, and G) show that the most common allele is significantly more frequent than the second most common allele (Fisher’s Exact tests, *p*-values given below the bracket (*p* < 0.0001 applies to both higher frequency alleles in A and C)). The asterisk (E) indicates that the second most common alleles, in Gombe and BPRC, have significantly different frequencies (Fisher’s Exact test, *p* = 0.033). Overall, the *Patr-B* frequency distributions for Gombe and BPRC do not differ significantly (*χ^2^* test) (In this analysis, the frequencies of the two rarest Gombe alleles were combined so as to give equal allele numbers for the two populations). The *HLA-B* distributions of the indigenous human populations do not differ significantly from the *Patr-B* distributions of the chimpanzee populations. The *HLA-B* distributions in urban human populations do differ significantly (*χ^2^* tests, *p* < 0.01) from the Patr-B distributions of the chimpanzee populations (In this analysis, only the three most common alleles were included individually; all other alleles were combined to equalize the number of alleles for the different populations). N: the number of individuals analyzed. Data for this figure are provided in S1 Data.

Eleven *Patr-B* alleles were identified in the Gombe chimpanzee population (S1 Table). Their distribution is consistent with Hardy-Weinberg equilibrium (*p* = 0.199, standard error (SE) = 0.02) (S1 Fig). Two alleles (Patr-B*22:04 and Patr-B*23:05) account for 58.8% of the total *Patr-B* alleles, having frequencies that are significantly higher than those of the nine low-frequency alleles (Fig 3A). Each community has nine or ten *Patr-B* alleles, with eight alleles being common to the three communities (Fig 3B–3D). Patr-B*22:04 and B*23:05 have high frequencies in the medium-sized northern community (Fig 3B) and the large central community (Fig 3C), whereas in the small southern community (Fig 3D), the B*22:04, B*23:05 and B*39:01 alleles are at lower, and comparable, frequencies.

In their number and relative frequencies, the Gombe *Patr-B* alleles are comparable to those of a captive *P. t. verus* population (Fig 3E) that was founded with 32 wild chimpanzees from Sierra Leone [41]. This captive population, formerly housed at the Biomedical Primate Research Center (BPRC) in the Netherlands, has ten *Patr-B* alleles: one at high frequency, four at intermediate frequency and five at low frequency. Certain small indigenous human populations in Africa (Fig 3F) and Asia (Fig 3G) have distributions of *HLA-B* alleles that are comparable to those of *Patr-B* in the Gombe and BPRC chimpanzee populations. In contrast, modern urban populations from Africa (Fig 3H), Europe (Fig 3I), and other continents (not shown) have higher numbers of *HLA-B* alleles, with more even frequencies. In the Gombe chimpanzees, the distribution of *Patr-B* alleles is significantly different from that of neutral markers, such as autosomal microsatellites and the mitochondrial D loop (*χ^2^* tests, *p* < 0.001 (D2S1326, D2S1333), *p* < 0.005 (D9S922, hypervariable D loop) (S2 Fig). Thus the distribution of *Patr-B* alleles does not reflect the overall genetic diversity of the Gombe population, suggesting that the *Patr-B* distribution is a consequence of selection.

Seven of the eleven Gombe *Patr-B* alleles are newly identified (submitted to the Genbank and IPD databases, with accession numbers provided) (S5 Fig). Four are identical to alleles found previously in captive chimpanzees (Patr-B*17:02, 17:03 [20]; Patr-B*22:03 (Genbank DQ539676); Patr-B*23:04 [45]). Fifteen out of the sixteen total nucleotide substitutions in the novel alleles are nonsynonymous, resulting in a change of the amino acid. This heavy bias towards changes that alter the composition of the Patr-B protein reflects the role of natural selection in generating and maintaining *Patr-B* polymorphism.

**Fig 4:**
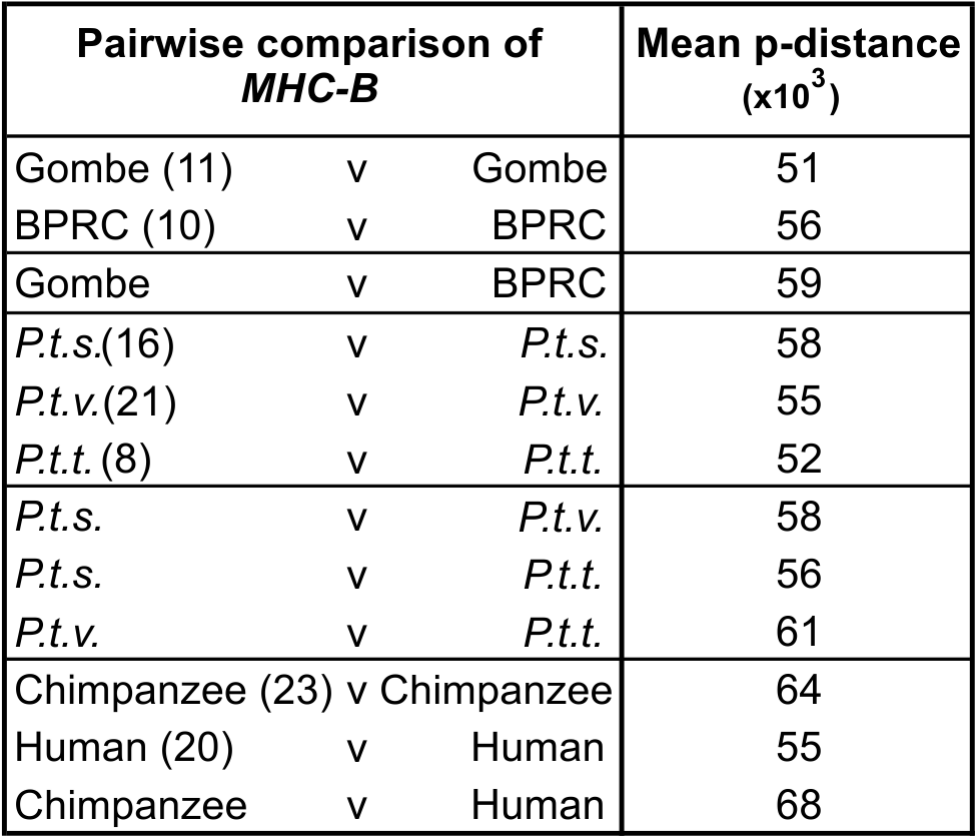
Sequence diversity of the polymorphic exons of chimpanzee and human *MHC-B* alleles. Pairwise nucleotide differences (p-distances) in the sequences of exons 2 and 3 were calculated for each set of comparisons listed. “*P.t.s.*” is *P. t. schweinfurthii*; “*P.t.v.*” is *P. t. verus*; “*P.t.t.*” is *P. t. troglodytes*. The number of alleles in each set being compared is given in parentheses. Histogram distributions and statistics are given in S4 Fig. The alleles used to generate the p-distances are listed in S6 Fig, and their sequences are included in S2 Data.

Comparison of *Patr-B* sequences from three chimpanzee subspecies (*P. t. schweinfurthii, P. t. verus,* and *P. t. troglodytes*) shows that each allele has been found in only one subspecies (S3 and S6 Figs). This phenomenon is exemplified by the Gombe *P. t. schweinfurthii* (Fig 3A) and BPRC *P. t. verus* (Fig 3E) populations, which have no *Patr-B* allele in common. However, reflecting their common origins, different subtypes of *Patr-B* alleles have been found across the subspecies. For example, Patr-B*23:01 is a *P. t. verus* allele, B*23:02 a *P. t. troglodytes* allele, and B*23:03, 22:04 and *22:05* are *P. t. schweinfurthii* alleles (S3 Fig). Of the five broad clades of *Patr-B* alleles, four (b1, b2, b3, and b5) are well represented in the Gombe population, whereas the b4 clade appears specific to *P. t. verus* (S3 Fig). Comparison of *MHC-B* sequences (S4 Fig) shows that the mean pairwise nucleotide difference for the Gombe population is 88% of that observed for the *P. t. schweinfurthii* subspecies, 80% of that observed among all chimpanzees, and 75% of that observed in comparisons between human and chimpanzee *MHC-B* (Fig 4). Although small in size, the Gombe population has a portfolio of *Patr-B* alleles that embraces a considerable proportion of the total chimpanzee *Patr-B* diversity.

### The Patr-B Allotypes Enriched in Gombe Chimpanzees Are Not KIR Ligands

**Fig 5:**
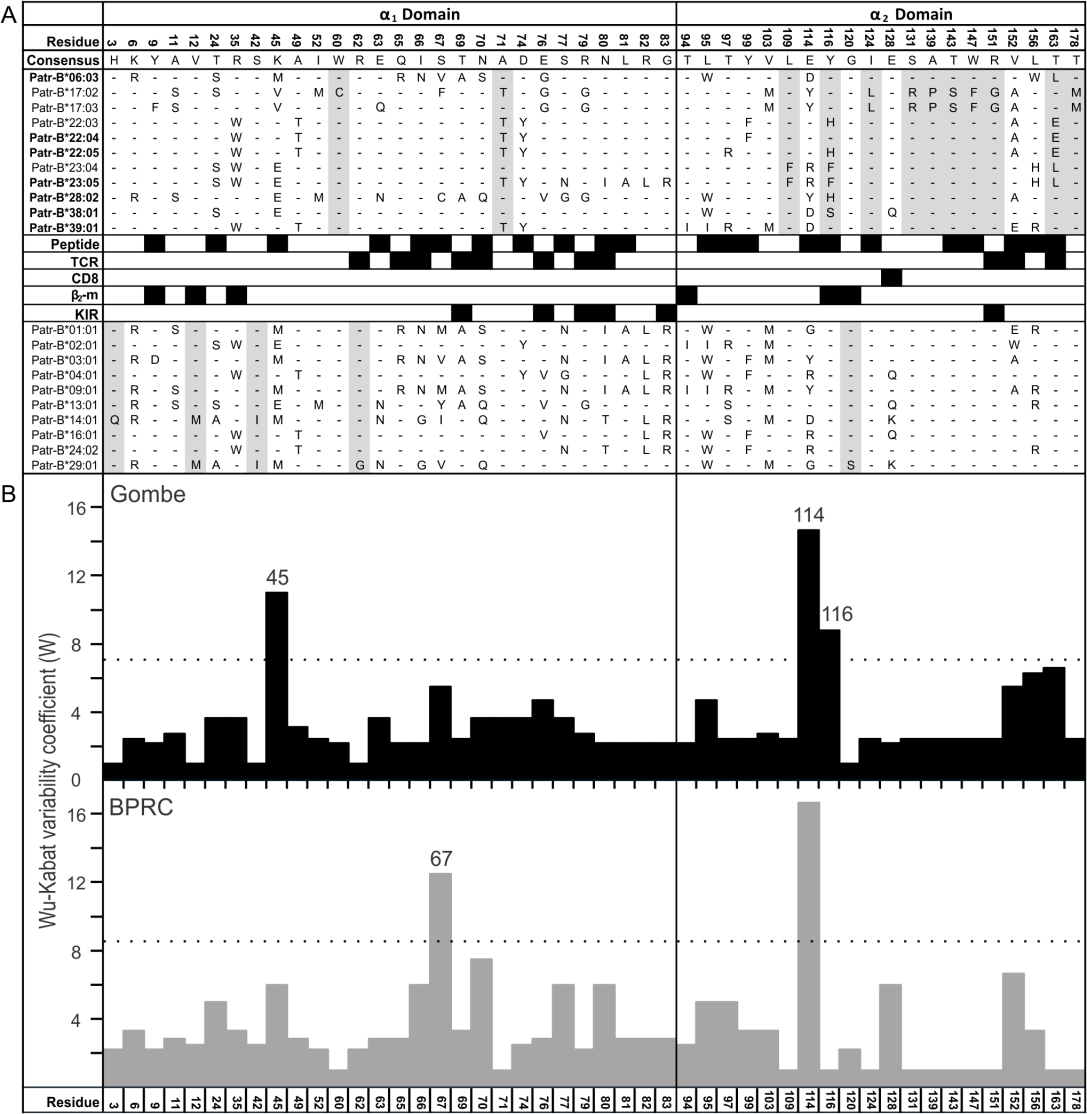
Extensive amino acid sequence polymorphism in the Patr-B allotypes of the Gombe and BPRC chimpanzees. (A) Displayed are the amino acid sequence differences that distinguish the α_1_ and α_2_ domains of Gombe *P. t. schweinfurthii* (upper group) and BPRC *P. t. verus* (lower group) Patr-B allotypes. Only polymorphic positions are included. Identity to the consensus is denoted by a dash. For amino acid differences from the consensus, the residue is shown. The black-filled boxes between the two sets of sequences show which residues contribute to binding sites for peptide, T cell receptor (TCR), CD8 T-cell coreceptor (CD8), KIR and the invariant β_2_-microglobulin subunit (β_2_-m) of the MHC class I molecule. In bold are the names of “novel” alleles, which were discovered in the Gombe population. Positions highlighted in gray are polymorphic in one population but not the other. (B) Histograms showing the value of the sequence variability coefficient, W, for the Gombe (upper) and BPRC (lower) Patr-B allotypes. The histograms are in vertical alignment with the sequence alignment in (A). For each population, the horizontal dashed lines mark the value for W that is twice the mean W value for all polymorphic residues in the population.

The distribution of amino acid substitutions within the α_1_ and α_2_ domains of the eleven Gombe Patr-B allotypes is characteristic of highly polymorphic MHC class I molecules that present peptide antigens to CD8 T cells. The polymorphic residues are principally those that determine peptide-binding specificity, with a lesser contribution from residues that engage αβ T cell receptors. Comparison of the patterns of sequence diversity in the Gombe and BPRC populations shows differences in the extent of diversity at several of the functionally important positions, notably residues 45, 67, 114, and 116 (Fig 5). These differences indicate that the two populations, of different subspecies, have been subject to different selective pressures from viral and other intracellular infections. One known difference is that SIVcpz is endemic to wild *P. t. schweinfurthii* populations but has not been found in wild *P. t. verus* populations [13].

**Fig 6:**
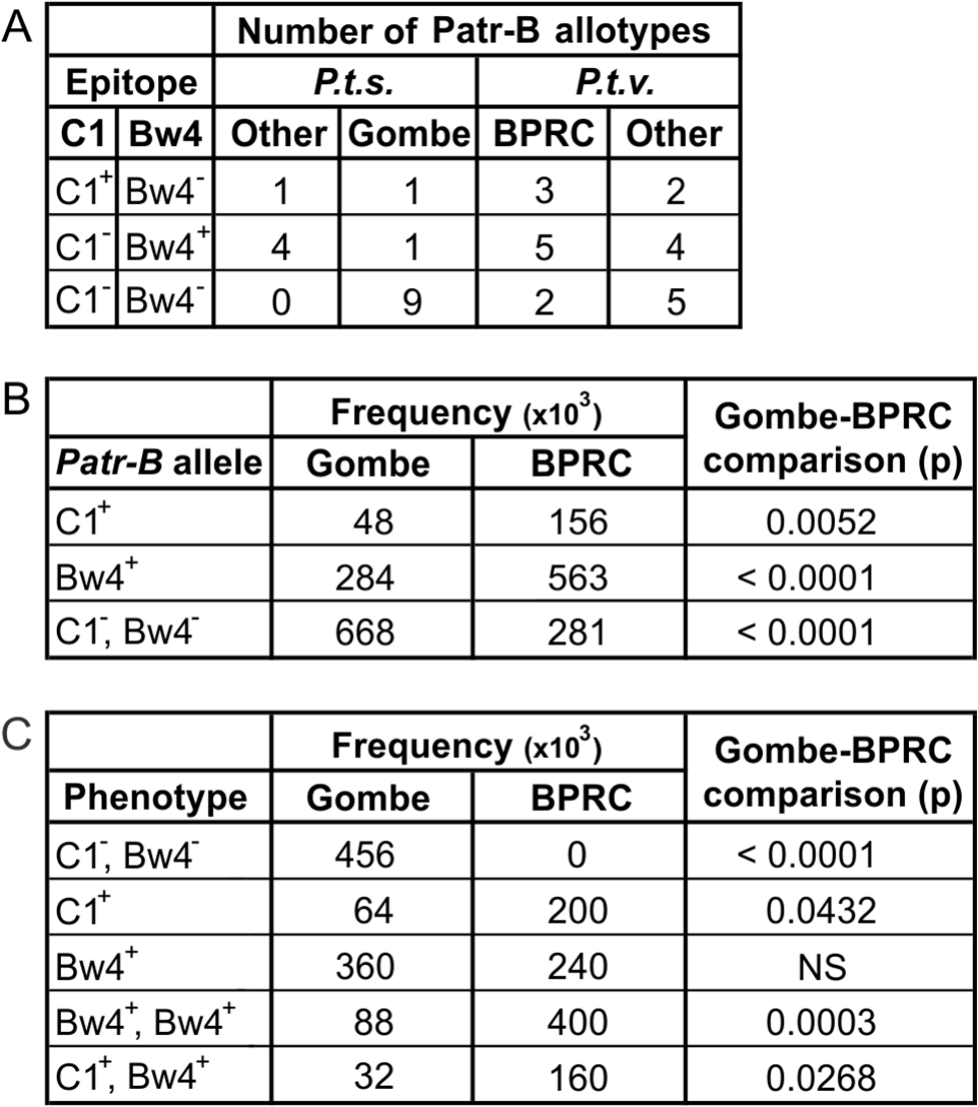
Significant differences in the Gombe and BPRC Patr-B allotypes bearing C1 and Bw4 KIR ligands. Plus and minus superscripts following C1 and Bw4 indicate presence or absence of the epitope. (A) Gives the distribution of the C1 and Bw4 epitopes recognized by KIR among the known 16 *P. t. schweinfurthii* (*P.t.s.*) and 21 *P. t. verus* (*P.t.v.*) *Patr-B* allotypes. The Gombe *P. t. schweinfurthii* allotypes are considered as a separate group from the “Other” *P. t. schweinfurthii* allotypes that are not present in the Gombe population. Likewise, the BPRC *P. t. verus* allotypes are considered as a separate group from the “Other” *P. t. verus* allotypes that are not present in the BPRC population. (B) Gives the frequencies of the *Patr-B* alleles encoding C1 and Bw4 epitopes in the Gombe and BPRC chimpanzee populations. Frequencies were transformed to integers by multiplying times 10^3^. Statistical significance was tested using Fisher’s exact tests, and *p*-values are provided (Gombe-BPRC comparison (p)). (C) Gives the phenotype frequency of the C1 and Bw4 epitopes in the Gombe and BPRC populations. Statistical significance was tested using Fisher’s exact tests and *p*-values are provided (Gombe-BPRC comparison (p)).

A further difference between the Gombe and BPRC Patr-B allotypes is their capacity to be recognized by NK cell receptors of the KIR family (Fig 6). KIR recognition of MHC-B is determined by sequence motifs at positions 76–83 in the α_1_ helix. Different motifs form the Bw4 and C1 epitopes, which are ligands for lineage II and III members of the KIR family, respectively [6]. Other sequence motifs at positions 76–83 of MHC-B do not permit KIR interaction. In the Gombe population, only two of the eleven Patr-B allotypes are predicted to be KIR ligands: Patr-B*28:02 that has the C1 epitope and Patr-B*23:05 that has the Bw4 epitope (Fig 6A). In contrast, all five of the other known *P. t. schweinfurthii* Patr-B allotypes, that are not present in the Gombe population, have either the C1 or the Bw4 epitope. Equally striking was that eight of the ten Patr-B allotypes in the BPRC population are predicted to be KIR ligands: three have the C1 epitope and five have the Bw4 epitope. The difference between Gombe and BPRC in the proportion of Patr-B allotypes predicted to be KIR ligands is statistically significant (Fisher’s Exact Test, *p* = 0.007). The frequencies of C1-bearing and Bw4-bearing Patr-B allotypes were, respectively, 3.3 and 2.0 fold higher, in the BPRC chimpanzees than in the Gombe chimpanzees (Fig 6B). A significant consequence of these differences is that all BPRC individuals have a Patr-B allotype that is a KIR ligand, whereas that is only the case for just over half (54%) of the Gombe chimpanzees (Fig 6C). Consequently, the Gombe chimpanzees have less capacity to modulate NK cell immune function via KIR interactions with Patr-B. These differences are a further reflection that the two populations have been subject to different selective pressures from viral and other intracellular pathogens.

### Gombe Patr-B*22 Subtypes Are Associated with SIVcpz Infection

**Fig 7:**
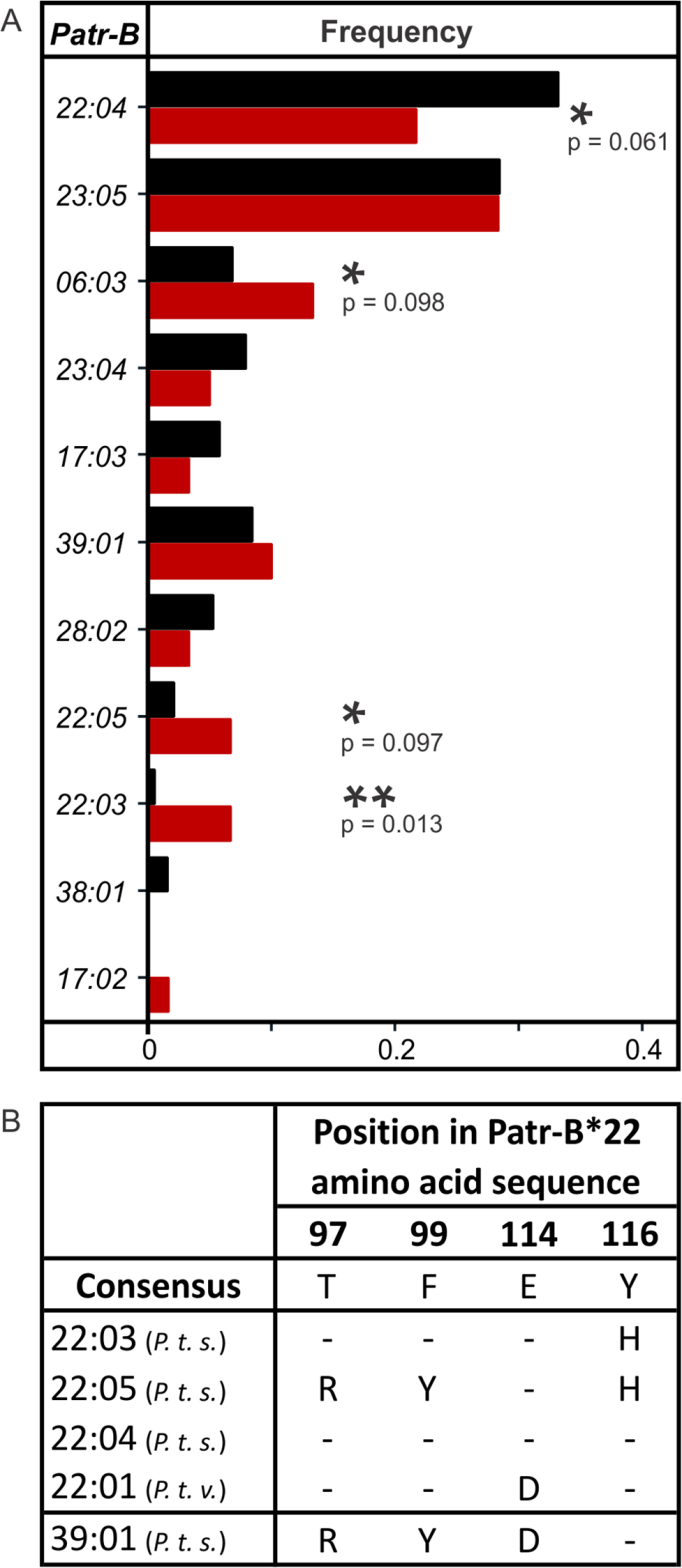
In the Gombe chimpanzees, SIVcpz infection correlates with increased frequencies for three *Patr-B* alleles. (A) Comparison of *Patr-B* allele frequencies in SIVcpz-infected and uninfected Gombe chimpanzees. The Patr-B*06:03, B*22:03, and B*22:05 alleles are at elevated frequency in the SIVcpz-infected chimpanzees. In contrast, the frequency of Patr-B*22:04 is lowered in the SIVcpz-infected chimpanzees. Asterisks denote differences between the SIVcpz-infected (red) (N = 30) and uninfected (black) (N = 95) subpopulations (Fisher’s Exact tests, *p*-values given underneath the asterisks *p < 0.1 and **p < 0.05). The frequencies for this figure are provided in S3 Data. (B) Sites of amino acid difference that distinguish Patr-B*22 subtypes. “*P.t.s.*” is *P. t. schweinfurthii*; “*P.t.v.*” is *P. t. verus*. Patr-B*39:01 is included because it has structural similarities with Patr-B*22, including an α_1_ domain that is identical to that of Patr-B*22:03 and B*22:05.

Thirty of the 125 Gombe chimpanzees included in this study are infected with SIVcpz. Comparison of the *Patr-B* alleles of infected and uninfected individuals shows no correlation with *Patr-B* homozygosity or heterozygosity (Fisher’s Exact test, *p* = 0.46). Three of eleven *Patr-B* alleles-Patr-B*06:03, B*22:03, and B*22:05-are over-represented in SIVcpz-infected chimpanzees (Fig 7A). Of these, Patr-B*22:03 and B*22:05 are low-frequency alleles: four of eight individuals having B*22:05, and four of five individuals having B*22:03, are SIVcpz-infected. Closely related to Patr-B*22:03 and B*22:05 is the high-frequency B*22:04 allele that is under-represented in the infected chimpanzees (Fig 7A). Thus, Patr-B*22:04 might confer some protection from SIVcpz infection. The Gombe B*22:03, B*22:04, and B*22:05 allotypes, as well as the *P. t. verus* B*22:01 allotype are all distinguished by sequence motifs at positions 97, 99, 114, and 116 in the α_2_ domain. SIV-associated B*22:03 and B*22:05 both have histidine at position 116, whereas B*22:04 has tyrosine (Fig 7B). Polymorphism at position 116 in human HLA-B is associated with significant host control of HIV-1: tyrosine, phenylalanine, and aspartic acid at this position are associated with patients who control their HIV-1 infection, whereas serine and leucine are associated with patients whose HIV-1 infection progresses to AIDS [46]. Although B*22:03 and B*22:05 are both correlated with SIVcpz infection, the effect is greater for B*22:03. That these allotypes differ only at positions 97 and 99 implies that polymorphism at these positions, which make contact with the peptide in the binding pocket (Fig 5A), modulates susceptibility or resistance either to infection or disease (Fig 7B).

The novel Gombe allotype, Patr-B*39:01, and the four Patr-B*22 allotypes have identical α_1_ domains (encoded by exon 2) (Fig 5A). The Patr-B*39:01 and B*22 α_2_ domains differ at five positions (94, 95, 103, 152, and 156) (Fig 5A), but at the four positions distinguishing B*22 allotypes (97, 99, 114, and 116), B*39:01 has a unique combination of the B*22 residues (Fig 7B). Thus, B*39:01 has similarities with B*22 in both the α_1_ and α_2_ domains. Although its frequency does not differ between SIVcpz-infected and uninfected chimpanzees (Fig 7A), Patr-B*39:01 has other associations with SIVcpz through its increased frequency in the SIVcpz-afflicted southern community and correlation with increased viral load (VL), as discussed below. These results further implicate residues 97, 99, 114, and 116 as key modulators of susceptibility or resistance.

### SIV-Associated Patr-B*06:03 Is Part of an Exceptional Clade of Ape and Human MHC-B

**Fig 8:**
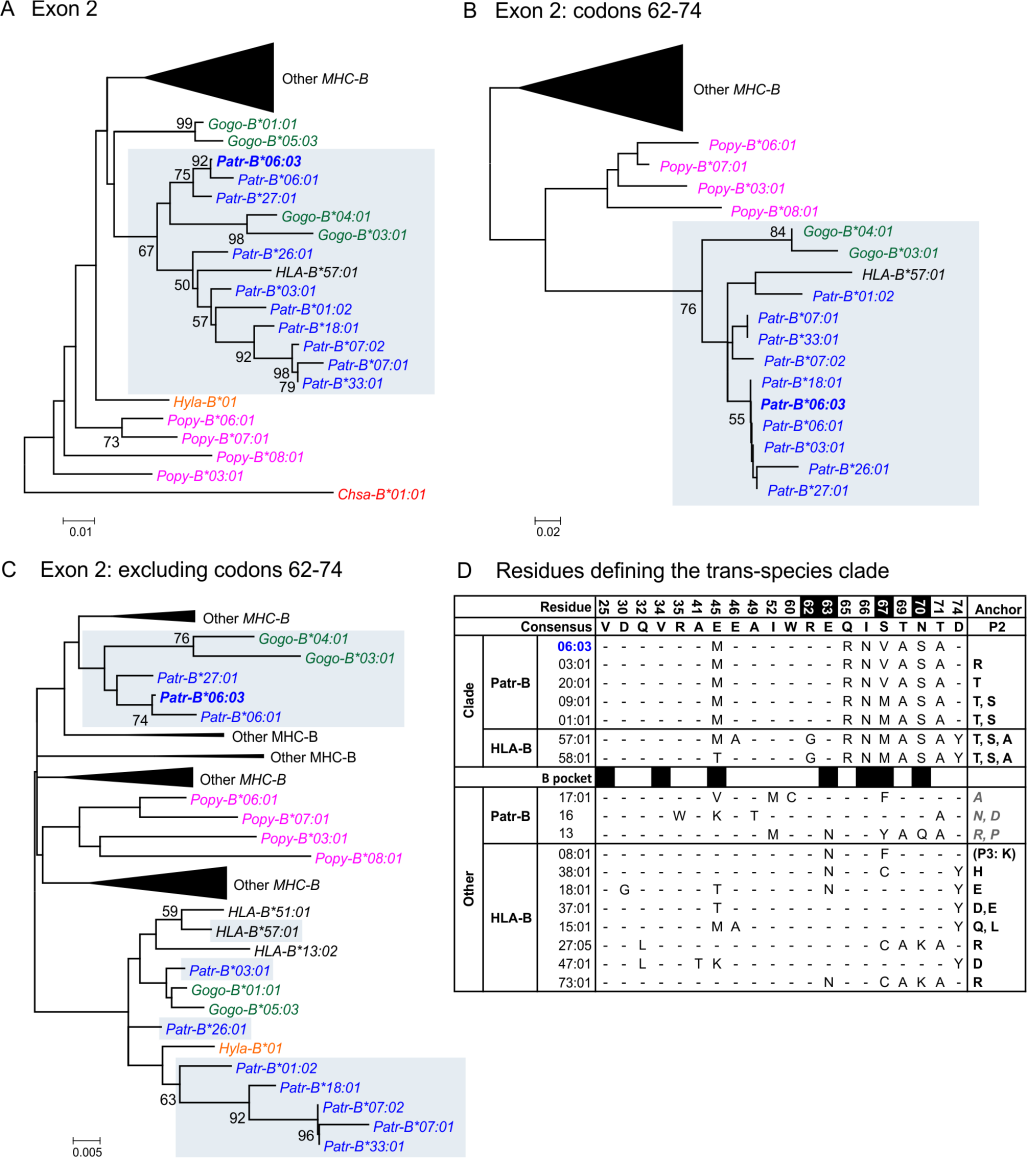
SIVcpz-associated Patr-B*06:03 is related in structure and evolution to HLA-B*57:01. Shown is a neighbor-joining phylogenetic tree for the exon 2 sequences of representative *MHC-B* alleles and the four novel, nonrecombinant Gombe *Patr-B* alleles (A). An African green monkey (*Chlorocebus sabaeus*) *MHC-B* is the outgroup (*Chsa*, red) in panel A. The gray shaded box denotes the unique clade containing Patr-B*06:03 (bold blue). This clade is maintained in trees constructed from the sequence of codons 62–74 (B), but not in trees constructed from exon 2 sequences with codons 62–74 deleted (C). This shows that codons 62–74 are the basis for the clade and the relatedness of its members. Nodal bootstrap values are based on 1,000 replications, and only values with at least 50% support are shown. Nodes that were collapsed are represented by large, black triangles. Chimpanzee alleles (*Patr*) are in blue. Also included are human (*HLA*, black), orangutan (*Popy*, pink), western gorilla (*Gogo*, green), and white-handed gibbon (*Hyla*, orange) *MHC-B*. Bonobo (*Papa*) sequences were also included in the analysis, but are within the collapsed nodes. S7 Fig gives the full trees with all the taxa, and the sequences are included in S2 Data. (D) The trans-species clade containing Patr-B*06:03 (bold blue) and HLA-B*57:01 is defined by an extensive sequence motif covering residues 25–74 of the α_2_ domain. The sequences of representative MHC-B allotypes that are in the clade, or not in the clade (Other), are compared. Only positions of difference are shown. Identity to the consensus is shown by a dash, with differences from the consensus being shown by the amino acid substitution. Black-filled boxes show residues of the B pocket that bind the anchor residue at position 2 (P2) of peptide antigens [53,54]. Under “Anchor” and “P2” are listed the anchor residues at P2 that are predicted to bind to each MHC-B allotype (compiled from the SYFPEITHI database of MHC ligands and peptide motifs, http://www.syfpeithi.de/ [55] and de Groot et al. (2010) [41]). Patr-B P2 residues that were inferred from known ligands are in gray italics. HLA-B*08:01 is distinguished by having a P3 anchor residue [55]. Residue positions that have been associated with control of HIV are indicated by white numbers on black background [46,56].

Patr-B*06:03 has a higher frequency than B*22:03 and B*22:05, being carried by 20 individuals of whom seven are SIV-infected. Much of the phylogenetic tree of hominoid *MHC-B* exon 2 sequences is shallow and consists mainly of clusters of closely related, species-specific alleles (S7A Fig). By contrast, Patr-B*06:03 is part of an unusual clade that has deep structure and trans-species character (Fig 8A). Included in the clade are eight *Patr-B*, two gorilla *Gogo-B* alleles, and human HLA-B*57:01. For HIV-1-infected individuals, HLA-B*57:01 provides protection being strongly associated with reduced VL and delayed progression to AIDS [19,47–52]. The integrity of the trans-species clade is preserved in phylogenetic trees made from codons 62–74 of exon 2 (Fig 8B) but not in trees constructed from exon 2 sequences lacking codons 62–74 (Fig 8C). This result shows that sequence polymorphisms within residues 62–74 of the α_1_ domain define the trans-species clade. Comparing representative MHC-B allotypes that are in the clade with MHC-B allotypes that are not shows that the key substitutions are at positions 65, 66, 67, 69, 70, and 71 (Fig 8D). These residues are all in the amino terminal half of the α_1_ helix, and many of them contribute to the B pocket of the peptide-binding groove [53,54]. This pocket binds the side-chain of the critical anchor residue at position 2 (P2) of peptide antigens [54]. The B pockets of MHC-B molecules in the exceptional trans-species clade are characteristic of those that bind peptides with either serine or threonine as the anchor residue [41,55].

In the Gombe population, Patr-B*06:03 is the only member of the trans-species clade. It is likely associated with protective effects, given its relatedness to HLA-B*57:01. The over-representation of Patr-B*06:03 in SIVcpz-infected individuals is consistent with them experiencing longer survival through delayed progression to an AIDS-like stage of disease. Supporting this hypothesis, residues 62–74 of Patr-B*06:03 and other members of the trans-species clade include four (62, 63, 67, and 70) of seven residues found by genome-wide association study (GWAS) to be most significant for HLA-B mediated control of HIV-1 infection [46,56] (Fig 8D).

### Patr-B*06:03 Is Associated with Reduced SIVcpz VL

HLA-B*57:01 correlates with longer survival of HIV-1-infected humans through its association with reduced VL [19,47–52]. The reduction in VL likely results from a fitness cost to viral replication associated with a frequent mutation that occurs at position 242 in HIV-1 Gag (T242N) [57–60]. We therefore hypothesized that Patr-B*06:03 acts similarly in the control of SIVcpz. Because the capacity to amplify viral RNA (vRNA) by RT-PCR from the feces of captive primates correlates with the amount of virus in plasma [61], we used this as a proxy measure for the systemic VL of wild chimpanzees (see Materials and Methods for more detail). We thus determined the fraction of samples with successful amplification of vRNA from the feces of each SIVcpz-infected Gombe chimpanzee and then tested these values against the presence or absence of a particular *Patr-B* allele. Consistent with our hypothesis, successful RT-PCR amplification of vRNA occurred less frequently for chimpanzees having Patr-B*06:03 than from chimpanzees lacking Patr-B*06:03 (*p* = 0.005) (S9 Fig, and statistical summary in S10B and S10C Fig). The one Gag sequence that has been determined for SIVcpz isolated from a Patr-B*06:03-positive individual gave no evidence for a Gag T242N escape mutation, but it does have a T242S mutation (S11 Fig). The effect of this change, however, is unknown.

We also compared vRNA amplification rates for the Patr-B*22:03, 22:04, 22:05 alleles that, like Patr-B*06:03, differ in frequency between the cohorts of SIVcpz-infected and uninfected individuals (Fig 7A). Also tested were Patr-B*39:01, because of its similarity to B*22 alleles (Fig 5A and Fig 7B), and Patr-B*23:05 because of its high frequency within the population (Fig 3). Patr-B*39:01 correlates with increased success of vRNA amplification (*p* = 0.036), suggesting individuals with this allele have elevated VL, but the effect was only significant when B*39:01 was examined alone (S9 and S10B and S10C Figs). Patr-B*22:04 also has an association with higher VL when examined alone; however, the effect of Patr-B*22:04 is not significant when tested in combination with B*06:03 and B*39:01 (S10B and S10C Fig). Within the sensitivity range of the assay used, we saw no effects of Patr-B*23:05, Patr-B*22:03, and Patr-B*22:05. One explanation for the results is that these Patr-B allotypes have no effect on VL. Another is that they can reduce viral load to some extent but not at a level detected by our assay. Also possible is that we had inadequate power to detect an effect for the Patr-B*22:03 and *22:05* subtypes because they are present in only few individuals.

Because of the *Patr-B* associations with VL, we examined if longevity of SIVcpz-infected chimpanzees correlated with *Patr-B* alleles. We found no difference in the risk of death associated with Patr-B*06:03 using either more conservative or less conservative criteria (detailed in Materials and Methods and S1 Text) (S10D Fig). Neither was there any effect on longevity associated with any other SIVcpz-associated allele (S10D Fig). Similarly, in an analysis of all Gombe individuals, irrespective of SIVcpz infection, there was no association of *Patr-B* with longevity. Many of the SIVcpz-infected chimpanzees are still alive, so continued monitoring of the Gombe population should give more data and power to address these questions in the future. In summary, this analysis of SIVcpz RNA in feces suggests an association of Patr-B*39:01 with higher VL. The results are also consistent with Patr-B*06:03 acting to lower the VL of chimpanzees infected with SIVcpz. Thus, Patr-B*06:03 shares both structural and functional properties with human HLA-B*57:01.

### The Community with Highest SIVcpz Prevalence Has Experienced an Unstable Distribution of Patr-B Alleles

**Fig 9:**
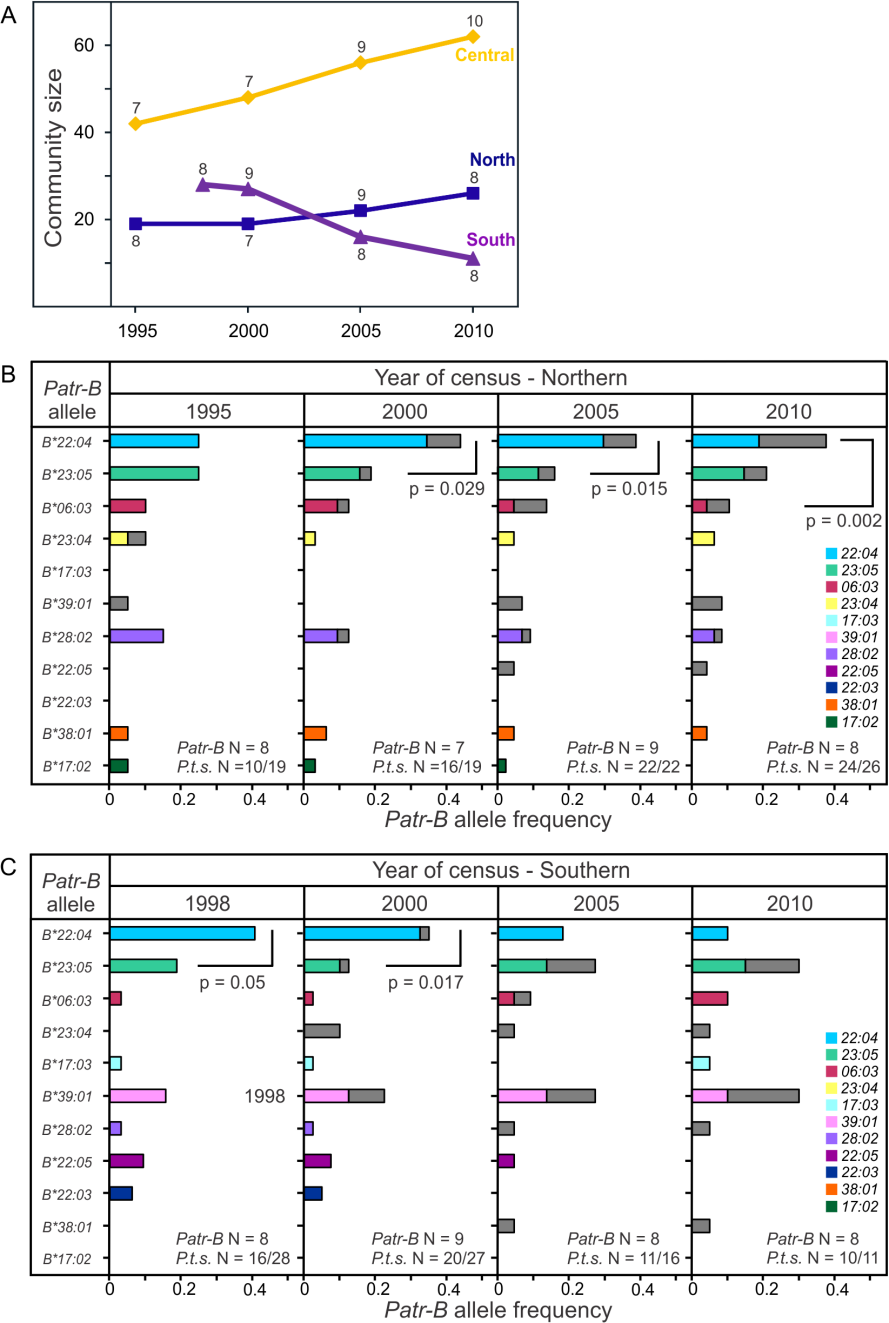
Population dynamics of *Patr-B* in the three communities of Gombe chimpanzees. (A) Shows changes in population size for the central (orange), northern (blue), and southern (purple) communities between the years 1995 and 2010. The earliest census year was 1998 for the southern community. The number of *Patr-B* alleles is given at each time point: diamonds for the central, rectangles for the northern, and triangles for the southern community. (B) Shows the temporal variation of *Patr-B* allele frequencies and the contribution of immigrant females and their offspring to the northern Gombe community. Partial gray shading of the frequency gives the contribution made by immigrant females who arrived during the study period and their offspring. *P.t.s.* N is the number of individuals that were genotyped for *Patr-B* out of the total number of individuals alive on the first day of the year. *Patr-B* N is the number of alleles present within the community. The brackets in 2000 and 2005 indicate a difference in the frequencies between the two most frequent alleles; the bracket between alleles in 2010 indicates that the highest frequency allele is significantly elevated compared to the third highest frequency allele (Fisher’s Exact tests, *p*-values given below the brackets). (C) Temporal variation of *Patr-B* allele frequencies and the contribution of immigrant females and their offspring to the southern community (Distributions for all three communities are provided together in S13 Fig). Partial gray shading of the frequency bars represents the contribution of immigrant females that arrived during the study period and their offspring. *P.t.s.* N is the number of individuals that were genotyped for *Patr-B* out of the total number of individuals alive on the first day of the year. *Patr-B* N is the number of alleles present within the community. The bracket in 1998 indicates a difference in the frequencies between the two most frequent alleles; the bracket between alleles in 2000 indicates that the highest frequency allele is significantly elevated compared to the third highest frequency allele (Fisher’s Exact tests, *p*-values given below the brackets). The overall frequency distribution for the southern community is significantly different between 2000 and 2005 (*χ^2^* = 11.343, *p* < 0.025) (all within-community frequency distributions (between time points) and comparison statistics for the total allele frequency distributions for all three communities are provided in S12 Fig). Community size and frequency data are provided in S4 Data.

**Fig 10:**
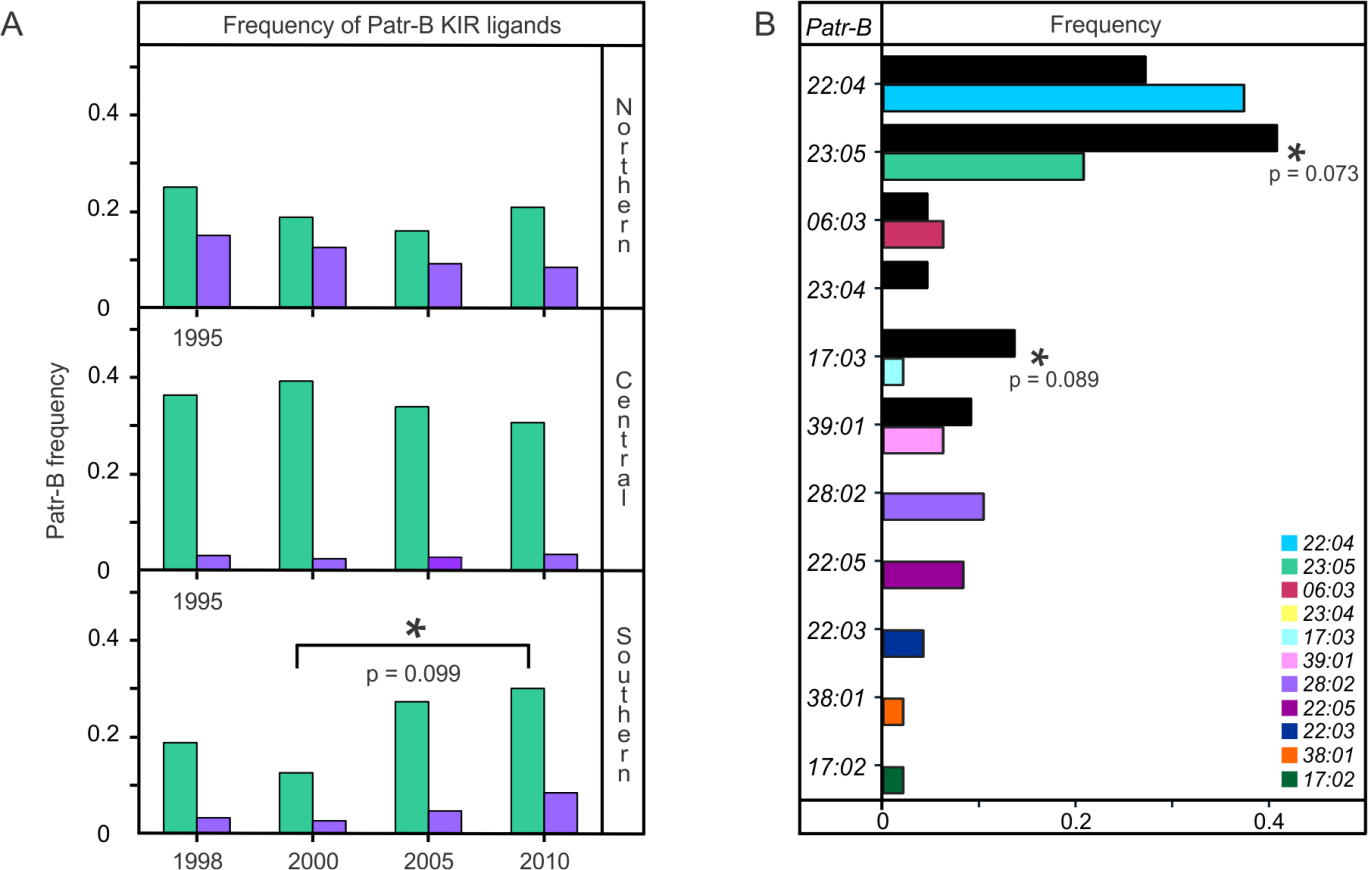
The frequency of the Bw4 KIR ligand is elevated among natal females and has increased in the southern community. (A) Patr-B*23:05 is the only Gombe Patr-B allotype that carries the Bw4 epitope, a ligand for KIR receptors of NK cells. Similarly, Patr-B*28:01 is the only Gombe Patr-B allotype that carries the C1 epitope, also a ligand for KIR. Shown are the temporal changes in the frequencies of Patr-B*23:05 (green bars) and Patr-B*28:01 (purple bars) in the three Gombe communities. In the northern and central communities, these frequencies were relatively stable, whereas in the southern community, there was a steady increase in the frequency of Bw4-bearing Patr-B*23:05 during 2000–2010 when the southern community was declining (Fisher’s Exact test, *p* = 0.099). Although not significant, a monotonic increase in the frequency of C1-bearing Patr-B*28:01 is also seen. The earliest census year was 1995 for the central and northern communities, and 1998 for the southern community. The community frequency data for these alleles are provided in S4 Data. (B) This compares *Patr-B* allele frequencies for natal (black bars) and immigrant (colored bars) female chimpanzees of the northern and central communities. The Patr-B*23:05 and Patr-B*17:03 alleles are enriched among natal females, but not immigrant females. Significance is denoted by asterisks (Fisher’s Exact tests, *p*-values are given beneath the asterisks). The frequency data are provided in S6 Data.

Since 1995, the northern and central communities of Gombe chimpanzees have increased in size, while the southern community has diminished because of death and emigration (Fig 9A and S14 Fig) [16,34]. A likely contributor to this decline is the higher frequency of SIVcpz infection in the southern community: 46% between 2002 and 2009, compared to <13% in the other communities [16,34]. A study which modeled SIVcpz and chimpanzee population dynamics suggested that female migration between communities could reverse such decline [16]. We therefore explored the temporal dynamics of *Patr-B* within the Gombe population with particular respect to female movements. Throughout the 1995–2010 period, the central community preserved a skewed distribution of alleles consisting of two high-frequency alleles (Patr-B*22:04 and *23:05*) and 5–7 low-frequency alleles (S12 Fig). The increased number of low-frequency *Patr-B* alleles was due to immigrant females (S13 Fig), coming mainly from the southern community but also the northern community (S14 Fig). In 1995, the northern community had a relatively even frequency of alleles, but subsequently Patr-B*22:04 rose to high frequency and was maintained largely because of immigrant females and the offspring they produced (Fig 9B and S12–S14 Figs). Most dynamic was the southern community. During 1998–2000, Patr-B*22:04 was a high-frequency allele, as in the northern and central communities, but its frequency subsequently decreased along with this community’s decline in size. Concurrently, Patr-B*23:05, the population’s only allotype bearing the Bw4 epitope recognized by KIR of NK cells, and B*39:01 increased in frequency and became the high-frequency alleles of the southern community (Fig 9C, Fig 10A, and S12 and S13 Figs). This development was mainly due to newly identified, and presumably immigrant, females and the offspring they produced in this unhabituated community (Fig 9C and S12–S14 Figs), even though Patr-B*23:05 was less frequent among immigrant than natal females (Fig 10B). Immigrant females also helped preserve allelic diversity by adding and replacing low-frequency alleles that had been lost through death and female emigration (S13 and S14 Figs). In summary, between 1998 and 2010, the smaller, SIVcpz-plagued southern community experienced major changes in its distribution of *Patr-B* alleles, whereas the distributions in the larger and healthier northern and central communities were relatively stable, with only minor fluctuations. Female migration was a major factor contributing to changes in *Patr-B* diversity.

### High-Frequency Alleles Were Maintained by Immigrants to the Northern Community and by Dominant, Fecund Individuals in the Central Community

**Fig 11:**
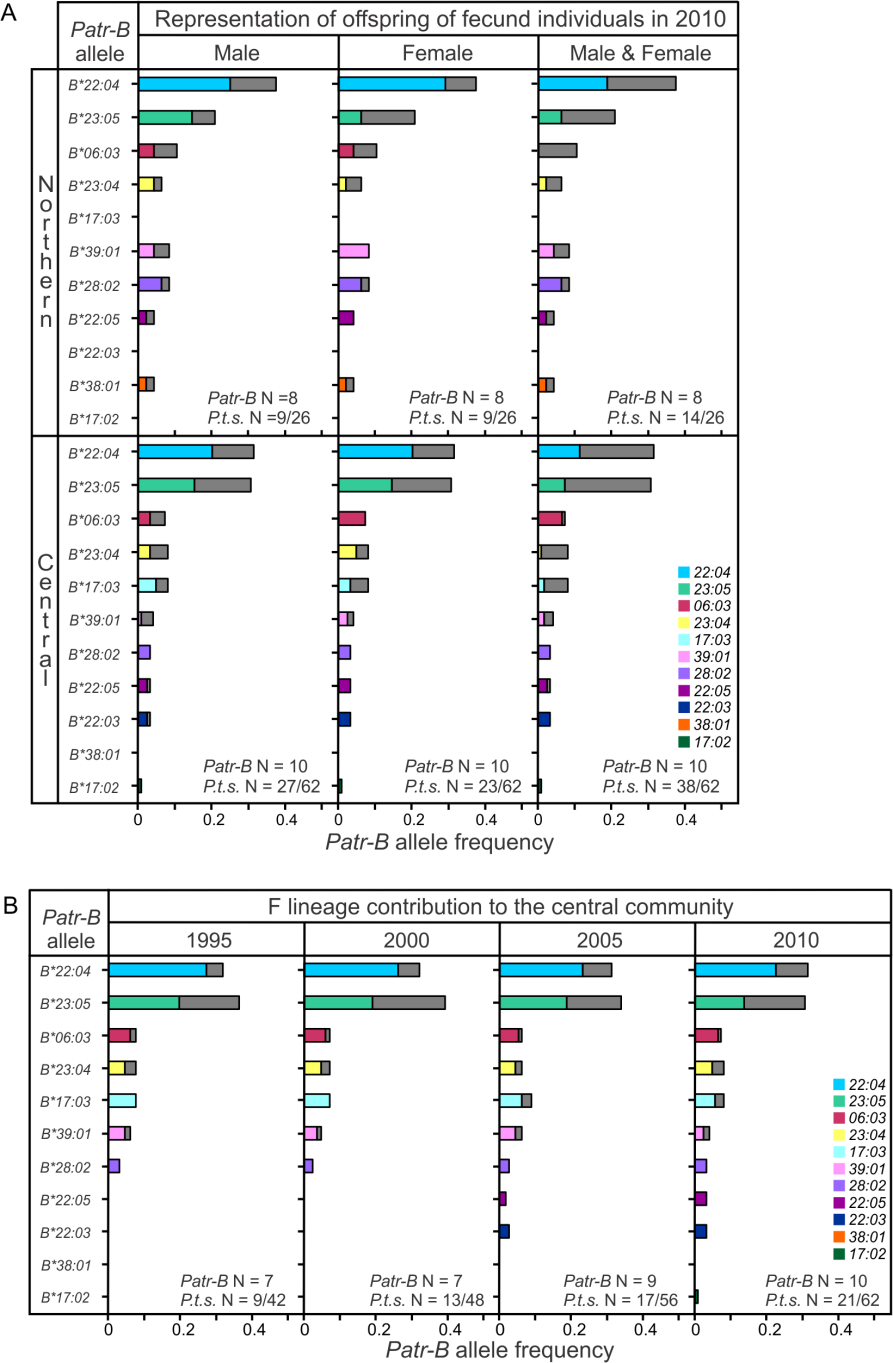
Fecundity of socially dominant chimpanzees is most influential in Gombe’s central community. (A) Contribution of the offspring of fecund individuals to the 2010 *Patr-B* allele frequencies of the northern and central communities. Partial gray shading of the frequency bars gives the allelic contribution of the offspring produced by fecund individuals. Fecund males (Male) were current or former alpha males. Fecund females (Female) in the central community produced at least twice the mean number of offspring. In the northern community, fecund females were defined as having more offspring than the mean. “Male and Female” combines the offspring produced by the fecund females for each community with either that of the top two producing fecund males for the central community, or that of the three alpha males for the northern community. *Patr-B* N is the number of alleles present within the community, while *P.t.s.* N is the number of offspring of fecund individuals in the population on the first day of the year. Frequencies are provided in S4 Data and S7 Data. (B) Shows how the matrilineal F lineage contributed substantially to the high frequency of Patr-B*23:05 in the central community. Partial gray shading of the frequency bars gives the contribution of F lineage individuals. *Patr-B* N is the number of alleles present within the community. *P.t.s.* N is the number of F lineage individuals in the population on the first day of the year. Frequency data are included in S4 Data.

In addition to an increased likelihood of death, SIVcpz infection is associated with reduced female reproductive success [37]. While both these effects can affect genetic diversity, as seen in the southern community, the relative stability of *Patr-B* in the other communities could be a consequence of reproduction that compensates for the deleterious effects of SIVcpz infection. We therefore explored the influence of reproductive success on *Patr-B* allele frequencies in the central and northern communities. For chimpanzees, reproductive success is socially driven since the most fecund individuals are usually socially dominant [36,62–65] (see Materials and Methods for detail). The central community maintained both B*22:04 and B*23:05 as high-frequency alleles throughout the study period. None of the seven dominant males in this community were SIVcpz-infected. In 2010, their offspring accounted for 36%–50% of the B*22:04 and B*23:05 high-frequency alleles. Moreover, the offspring of alpha males constituted 44% of the community (Fig 11A). The offspring of fecund, and high-ranking, females (some of which were also the offspring of alpha males) represented 36%–53% of the high frequency alleles and 37% of the community. None of the five fecund females tested (of the six, total) were SIVcpz-infected, and three were natal females, born into and staying in the community to reproduce. Together, the offspring of two males and six females accounted for 61% of the central community and 64%–76% of the two high frequency *Patr-B* alleles (Fig 11A). Thus, the dominance of two *Patr-B* alleles in the larger central community is a consequence of the reproductive success of a small number of fecund and socially-dominant males and females.

Since 2000, Patr-B*22:04 has been the only high-frequency allele in the northern community (Fig 9A and 9B). The offspring of the three alpha males known in this community contributed 33% of the Patr-B*22:04 allele frequency and represented 35% of the community in 2010 (Fig 11A). This contribution was comparable to that of the alpha males in the central community, despite two of the northern alpha males being SIVcpz-infected. Although none of the four fecund females tested (of five in total) were SIVcpz-infected, their contribution to the high-frequency allele was less than seen in the central community. Offspring of northern fecund females represented only 22% of the Patr-B*22:04 alleles, while representing 35% of the community in 2010. The combined offspring of fecund males and females in the northern community contributed 50% of the Patr-B*22:04 alleles (Fig 11A). Although substantial, this is less than the 67%–74% observed in the central community. Thus, the overall impact of fecund individuals was less pronounced in the northern community than in the central community. Instead, the high frequency of Patr-B*22:04 in the northern community was more influenced by immigrant females. Overall, all the known immigrants to the northern community and their offspring contributed 50% of the Patr-B*22:04 alleles in 2010, compared to 33% of B*22:04 in the central community (Fig 9B and S13 and S14 Figs). In addition, none of the five fecund females of the northern community were known to be natal females, and three were immigrants. In sum, under low selective pressure from SIVcpz, the *Patr-B* allele distributions of the northern and central communities were driven by the reproductive success of a small number of chimpanzees. These comprised mostly immigrant females to the northern community and fecund and socially dominant males and females in the central community.

One central community matriline produced three of the last seven alpha males (S15 Fig) and two of the six most fecund females. In 2010, this F matriline contributed 55% of the Patr-B*23:05 allele frequency and represented 34% of the 2010 population (Fig 11B). Without this contribution, the frequency distribution of the central community would be more like that of the northern community, where Patr-B*22:04 dominates. Thus, the F lineage specifically contributed to the high Patr-B*23:05 frequency, whereas the offspring of fecund individuals contributed to the high frequency of both Patr-B*22:04 and B*23:05.

In the central community, the effects of fecundity are further observed in the *Patr-B* frequencies of the offspring of fecund females compared to other females (S16 Fig). In addition to a difference in the total distribution (*χ^2^* = 8.202, *p* < 0.05), the two Patr-B allotypes bearing epitopes recognized by KIR of NK cells also differ: Patr-B*23:05, with a Bw4 epitope, is more frequent among the offspring of fecund females (Fisher’s Exact test, *p* = 0.063), while Patr-B*28:01, with a C1 epitope, is more frequent among the offspring of less fecund females (Fisher’s Exact test, *p* = 0.078) (S16 Fig). The SIVcpz-associated allele, Patr-B*06:03, is significantly elevated in the offspring of non-fecund females (Fisher’s Exact Test, *p* = 0.021). No such differences were observed in the offspring of males. Neither were such associations seen in the northern community, where fecundity differences appear less strong. However, 70% of the northern community’s Patr-B*23:05 alleles were contributed by the offspring of fecund females (Fig 11A). These observations further raise the possibility that B*23:05, the only Bw4-bearing allotype, directly influences fecundity.

In summary, reproductive success was the dominant influence on *Patr-B* allele distributions in the communities less pressured by SIVcpz infection. Immigrant females had most influence in the northern community, whereas fecund and socially high-ranking males and females dominated reproduction in the central community. In particular, the dominance of one matriline was responsible for the central community’s unique high-frequency allele.

## Discussion

The population of *P. t. schweinfurthii* chimpanzees in Gombe National Park, Tanzania has been systematically studied for more than 50 years [33]. This unique body of knowledge provided an unprecedented opportunity to study longitudinal population dynamics of the MHC in the living species most closely related to humankind. Such studies, which are critical for understanding evolution of the MHC and the selective forces that drive it, have not been performed in humans, where population studies are dominated by static, single time point characterizations, and disease-association studies are almost always retrospective and incompletely controlled. In this investigation, we focused on *Patr-B*, the ortholog of *HLA-B,* the most polymorphic gene in the human genome [8,20,21]. The Gombe chimpanzees are naturally infected with SIVcpz [16,37–39], permitting assessment of the impact of infection upon *Patr-B* polymorphism in each of the three communities of the Gombe population.

Although small by human standards, the Gombe population of approximately 125 chimpanzees maintains a diversity of 11 *Patr-B* alleles, eight being present in each of the three communities. In size, the central community increased considerably, and the northern community increased moderately during the last 15 years. Female immigration contributed to this expansion of these two communities [16,34]. In contrast, during this same period, the southern community steadily declined, which is in part due to this community’s higher prevalence of SIVcpz infection [16]. The relative success of the three communities is reflected in the frequency distributions of their *Patr-B* alleles. In the northern and central communities, the *Patr-B* distributions have remained relatively stable and similar, both consisting of a small number of high-frequency alleles and a larger number of low-frequency alleles. The high-frequency allele of the northern community derives predominantly from immigrant females who were reproductively successful, whereas in the central community, they derive from socially dominant and fecund males and females. Their reproductive success could have countered the deleterious effects of increased mortality and decreased female fecundity associated with SIVcpz infection and in this way maintained the viability of these communities. That is not the case for the southern community, which has lost population through the combined effects of SIVcpz infection and emigration [16]. Consequently, the southern community is experiencing a severe population bottleneck and striking changes to its *Patr-B* distribution. Improving the prospects of the southern community are immigrants who prevented even greater reductions in population size and *Patr-B* diversity. Thus, immigration and fecundity are important factors in the maintenance of community size and *Patr-B* diversity.

Analysis of the total Gombe population shows that three *Patr-B* alleles (Patr-B*06:03, Patr-B*22:03, and Patr-B*22:05) have significantly higher frequencies in the SIVcpz-infected chimpanzees than in the uninfected chimpanzees. Of these, the Patr-B*06:03 allele is a member of an exceptional trans-species clade of *MHC-B* alleles that is represented in chimpanzees, gorillas, and humans [41,66]. This clade includes human HLA-B*57:01, the *HLA-B* allele that has the strongest association with control of VL and delayed progression of HIV-1 infection to AIDS [19,47–52]. Underlying this protective effect are the actions of cytotoxic CD8 T cells [47]. These T cells recognize peptides derived from the HIV-1 Gag protein that are presented by HLA-B*57:01 on the surface of cells infected with HIV-1 [19,57,58], resulting in the killing of infected cells. In HIV-1-infected individuals with HLA-B*57:01, virus particles with mutations that enable them to escape such recognition often have a reduced capacity to replicate, thereby reducing VL [58–60]. For SIVcpz-infected chimpanzees, Patr-B*06:03 has a similar protective effect as that achieved by HLA-B*57:01 for HIV-1-infected humans. Using a qualitative PCR-based assay for detection, we find that vRNA is less abundant in the feces of SIVcpz-infected chimpanzees who carry Patr-B*06:03 than in the feces of chimpanzees lacking Patr-B*06:03. This correlation, itself, does not prove that Patr-B*06:03 causes reduced production of virus. However, the ancient, 13 residue structural motif that it shares with HLA-B*57:01, and that determines which peptides are bound and presented, is independent evidence that Patr-B*06:03 plays a comparable role to HLA-B*57:01 in stimulating lentivirus-specific CD8 T cells.

Three Patr-B*22 subtypes are represented in the Gombe population. Of these, Patr-B*22:03 and Patr-B*22:05 are over-represented in the SIVcpz-infected chimpanzees, whereas the Patr-B*22:04 is under-represented. These correlations are consistent with B*22:04 providing some resistance to SIVcpz infection, whereas B*22:03 and B*22:05 are either neutral or confer susceptibility. At position 116 in the Patr-B sequence, Patr-B*22:04 has tyrosine compared to histidine in B*22:03 and B*22:05. For human HIV-1 infections, tyrosine 116 in HLA-B correlates with reduced VL and slower progression to AIDS, whereas histidine 116 has no protective effect [46]. These parallels point to tyrosine 116 in Patr-B*22:04 being protective, but not histidine 116, in B*22:03 and B*22:05. That Patr-B*22:03 more strongly correlates with SIVcpz infection than B*22:05 shows that residues 97 and 99, which distinguish the two subtypes, also influence SIVcpz infection. Residue 97 is spatially close to residue 116 (and they could therefore interact), and, in HLA-B, 97 is the residue most significantly associated with host control of HIV-1 infection [46,56]. As seen for Patr-B*22 subtypes, arginine 97 is more strongly associated with infection than threonine 97 [46,56]. Unlike Patr-B*06:03, the three Patr-B*22 subtypes have no detectable effect on VL as assessed by the amplification of vRNA from feces. This does not rule out the possibility of more subtle VL differences that could be detected with the quantitative tests used for monitoring systemic VL in plasma. Alternatively, Patr-B*22:04 could protect by a different mechanism than reducing VL, or Patr-B*22:03 and Patr-B*22:05 could actively increase susceptibility to SIVcpz infection. However, while there are well-known associations between HLA-B and HIV-1 disease progression, correlations with susceptibility to becoming infected have not yet been found [19]. Patr-B*39:01, which shares structural similarities with Patr-B*22, correlates with increased VL and has increased in frequency during the decline of the southern community. Patr-B*39:01, which appears to promote the survival of infected chimpanzees without reducing VL, uniquely shares tyrosine 116 with Patr-B*22:04 and arginine 97 with Patr-B*22:05.

As cells of the innate immune response, NK cells have the potential to kill virus-infected cells and terminate infection at an early stage. NK cells can also provide responses at later stages of infection when the virus is outrunning the CD8 T cell response. HIV-1 and SIVcpz down-regulate the expression of MHC-B and MHC-A in the cells they infect [67]. This prevents virus-specific CD8 T cells from recognizing the infected cells but makes them more vulnerable to NK cell-mediated killing, which is triggered by loss of MHC class I expression. In particular, strong NK cell responses are made to cells losing expression of the C1 and Bw4 epitopes.

In acute human HIV-1 infection, there is a rapid NK cell proliferation [68], in which the expansion of particular NK cell subsets depends upon the presence of Bw4-bearing HLA-B allotypes [69]. Thus, the rise of Bw4-bearing Patr-B*23:05 in the southern community during its decline suggests that this allotype provides an advantage against SIVcpz by facilitating NK cell recognition of infected cells. The high frequency of B*23:05 in the central community could also have been due to selection for this function. The high frequency of B*23:05 is principally due to the reproductive success of a fecund and socially dominant lineage of chimpanzees in the central community, and it is significantly elevated among the offspring of its fecund females. In addition, natal females more frequently have B*23:05 than immigrant females. These characteristics could all result from a selective advantage of B*23:05 in fighting infection, or alternatively this allotype could have separable effects on immunity and reproduction. There is suggestion that Patr-B allotypes can play different roles in immunity and reproduction. The C1-bearing allotype, Patr-B*28:02, increased mildly in frequency in the southern community, but it is also enriched among the offspring of less fecund females in the central community. In addition, Patr-B*06:03 confers advantage in controlling SIVcpz infection but is inversely correlated with female fecundity (as seen in the central community).

Patr-B*06:03 and HLA-B*57:01 are part of an old and conserved clade that predates the evolutionary separation of human and African apes. This suggests that there has been a strong and consistent selective pressure that has maintained this clade. HIV-1 and SIVcpz are recent infections [13], but similar lentiviruses have likely been infecting African primates since sometime after the split of African and Asian monkey lineages [13] 6–10 million years ago [70]. Thus, these related viruses could have exerted selection pressure on human and African ape species throughout their history.

Despite their small sizes, the Gombe population and its constituent communities have maintained extensive *Patr-B* diversity, which is indicative of the strength of selection to maintain *MHC* diversity in small populations. In periods of health and population growth, as experienced by the northern and central communities, successful reproduction by socially dominant individuals gives rise to a stable distribution consisting of a few high-frequency alleles and a majority of low-frequency alleles. In a period of instability, infection, and declining population, as experienced by the southern community, there is selection for any alleles that give protection against the infectious disease. Small populations like the southern community can still be of relevance to evolution. Under selection, low-frequency alleles can quickly increase their frequency leading to major changes in the allele distribution and subsequent increase in the size and health of the population.

Well-illustrated by this study is how single events of recombination between *MHC-B* alleles can generate groups of MHC-B subtypes that offer different degrees of protection and susceptibility against an infecting pathogen. In both chimpanzees and humans, this is the major mechanism by which useful new variants are formed [20,66,71,72]. Reflecting the dynamic nature of the *MHC-B* gene, the majority of *Patr-B* alleles are specific to one subspecies of chimpanzee. It will therefore be of great interest to study *Patr-B* and other *MHC* genes in other subspecies of chimpanzee, as well in the second *Pan* species, the bonobo.

## Materials and Methods

Wild chimpanzee fecal collection was noninvasive and not deemed animal subjects research according to National Institutes of Health guidelines by the Stanford Administrative Panel on Laboratory Animal Care (APLAC). All observational data of the Gombe chimpanzees was approved by Tanzania National Parks, Tanzania Wildlife Research Institute, and Tanzania Commission for Science and Technology, as well as the Duke University Institutional Animal Care and Use Committee. Use of the Yerkes National Primate Research Center chimpanzee samples were approved under Stanford APLAC-9057.

### Study Site and Population

We studied the eastern *P. t. schweinfurthii* chimpanzee population of the Gombe National Park in northwestern Tanzania (Fig 2). Study of the Gombe chimpanzees began with Jane Goodall in 1960. Since 1977, three communities (the largest social unit formed by chimpanzees) with distinct territories have been recognized within the park [73]. These comprise the Kasekela (central) community, the Mitumba (northern) community, and the Kalande (southern) community. While males remain for life in the community where they were born, females typically emigrate to a new community when they reach sexual maturity; however, the extent of female migration is variable across communities and study sites [64,74,75].

The longest studied is the Kasekela community. Every day since 1973, one Kasekela individual has been followed and observed throughout the day, with a different community member being followed in turn on different days within a month. The size of the central community has varied from 37–62 individuals. Similar investigation of the northern community shows it has varied from 18–26 members since study began in 1997 [34,76]. The central and northern chimpanzees were both habituated to the presence of human observers [34,73]. The southern community, which is not habituated, has been monitored through routine searches (near daily) of the territory since 1999. Community size and composition has been estimated from opportunistic observations of chimpanzee sleeping nests and sightings of chimpanzees in which the number and the approximate age and sex of individuals are noted, as well as any visual identification of distinct individuals [16,34]. These observational methods are supplemented by genetic sampling from feces that are also collected opportunistically near nests and on trails. Since 1998, the community is estimated to have varied from 11–43 members, and it has steadily declined in size since 2002 [16,34].

### Sample Collection, Identification, and Paternal Pedigrees

Since 2000, noninvasive chimpanzee fecal samples have been collected in RNA*later* (Ambion) to preserve DNA and SIVcpz vRNA for sequencing (further described in S1 Text). They were collected as soon as possible after defecation in roughly equal volumes of feces and preservative (approximately 20–25 ml). Samples were then stored frozen in the field lab in Gombe National Park and later shipped to the United States and stored at −80°C. Using DNA extracted from feces [11,16,36,37,39,77], the visual identification of the chimpanzee sample donor by sample collectors was confirmed through several means (further described in the respective references): 1.) PCR-based sex determination [36,78], 2.) mitochondrial hypervariable D loop haplotyping (with further confirmation that samples from mothers and offspring shared the same haplotype) [36,40], and 3.) genotyping at 8–11 microsatellite loci [16,35,36]. The microsatellite genotypes were further used to determine paternity of offspring, whereby a father was the only male that could contribute half of the offspring’s alleles given the maternal genotype [16,36,79,80].

### DNA Extraction and Patr-B PCR and Sequencing

We extracted DNA for this study from fecal samples using the protocol described by Wroblewski et al. (2009) and the QIAamp DNA Stool Mini Kit (Qiagen) [36] (further described in S1 Text). Tissue samples were extracted using the DNeasy Blood and Tissue Kit (Qiagen). DNA was extracted from at least two independent samples per individual, whenever available. Because the DNA in feces consists of small, degraded fragments, PCR amplification was targeted to regions of the *Patr-B* gene having a size around 400 bp. Fragments of this size were successfully amplified in the microsatellite typing system applied previously to chimpanzee fecal DNA [36]. In separate amplifications we targeted exons 2 and 3 that encode the most polymorphic and functionally relevant parts of the Patr-B molecule. Primers were designed from conserved intronic sequences flanking these exons. PCR products were first directly sequenced with at least one of the amplification primers. Whenever a new allele was detected, or if results were unclear, the PCR products were cloned and then sequenced.

Known parent-offspring relationships were used to test the assigned genotypes and confirm that each individual had an appropriate combination of maternal and paternal alleles for exon 2 and exon 3 of *Patr-B* [16,36,79,80]. Inheritance patterns were also used to phase exon 2 and exon 3 sequences by determining which exon pairs assorted together. All novel *P. t. schweinfurthii Patr-B* alleles were confirmed by the observation of the allele in more than one individual or in at least two independent amplifications from the same individual.

### Identification of Core Hominid MHC-B Alleles

The exon 2 and 3 sequences for all *HLA-B* alleles in the IMGT-HLA database (http://www.ebi.ac.uk/ipd/imgt/hla/) (3.12.0, April 2013) and for all ape *MHC-B* alleles in the Immuno Polymorphism Database (IPD-MHC; http://www.ebi.ac.uk/ipd/mhc/) (1.9.0, July 2013) were compiled [21]. For each species a reduced set of “core” *MHC-B* alleles, from which all known alleles could be derived, was determined by the exclusion of all alleles that could be generated either by recombination or point mutation from the core alleles (S6 Fig).

### Phylogeny and Pairwise Differences between MHC-B Sequences

Neighbor-joining trees for *MHC-B* nucleotide sequences were constructed with MEGA v4.1 [81] using the Tamura-Nei method with pairwise deletion comparison and 1,000 replications. Pairwise sequence comparisons were conducted within and between species for chimpanzee *Patr-B* and human *HLA-B* alleles. Pairwise differences were also assessed between three chimpanzee subspecies (S6 Fig), for which *Patr-B* alleles only identified as being isolated from *Pan troglodytes* were excluded. No alleles from the *P. t. ellioti* subspecies have been defined. We calculated pairwise nucleotide distances for the sequences in each dataset using pairwise deletion and p-distance in MEGA v5.1 [82]. The difference between the means of allele pair sets was tested using unpaired *t* tests.

### Amino Acid Variability

Amino acid variability was determined for the Patr-B variants found within populations of chimpanzees using the Wu-Kabat Variability Coefficient [83]. The value (W) was calculated as N * k / n, where N is the number of sequences included in the analysis, and N is multiplied by k, the number of distinct amino acids at a given position. This product is then divided by n, the number of occurrences of the most common amino acid at that position.

### SIVcpz Detection, Viral Load Proxy, and Mortality Analyses

Fecal samples were screened by western blot for the presence of SIVcpz-specific antibodies as described previously [16,37,39]. A subset of samples from antibody-positive individuals were then subjected to nested RT-PCR amplification of vRNA to confirm SIVcpz infection and identify new viral variants. However, not all antibody-positive samples yielded PCR products [16,37,39] (S2 Table). Because previous studies of captive primates indicated a positive correlation between the ability to amplify vRNA from the feces and systemic VL of captive primates [61], we used the frequency of successful RT-PCR amplification as a proxy for VL in the wild Gombe chimpanzees. Fecal samples from SIVcpz-infected Gombe chimpanzees that were analyzed by RT-PCR were scored as vRNA positive or negative, regardless of which subgenomic region (pol, env, or nef) was amplified (S2 Table). Analysis was restricted to samples that were fecal western blot positive in order to ensure that samples were reasonably preserved and to exclude true as well as false negatives. We used Poisson regression to model the number of samples from which there was successful vRNA amplification out of the total number of samples tested (i.e., the success rate) according to *Patr-B* allele presence using SAS v9.3 (proc GENMOD). We did not include the community origin of the fecal sample as a covariate because Poisson regression analysis did not show an effect of community on the rate of successful vRNA amplification (S8 and S10A Figs).

Mortality analyses were conducted following those done for the northern and central communities in Gombe by Keele et al. (2009) [37], but we also included individuals from the southern community when life history data were available (further described in S1 Text). We used logistic regression with a complementary log-log link to model the age of death of SIVcpz-infected chimpanzees according to *Patr-B* allele presence, with age and sex as covariates, also using SAS v9.3 (proc GENMOD). We modeled two different data sets based on conservative and less conservative criteria for individuals’ SIVcpz status and dates of death. We also used the total population of both SIVcpz-infected and uninfected individuals to test for mortality differences according to *Patr-B* allele presence using the two data sets, further including SIVcpz status as a covariate.

### Study Period and Population Genetics

The demographic study period began in 1995 for the central and northern communities, which was the year for which we had DNA from at least 50% of the individuals who were alive in each habituated community on the first of the year. Comparable sampling of the southern community did not occur until 1998. The demographic composition of each community was assessed every five years through 2010 because five years is the mean interbirth interval for the Gombe chimpanzees [65,84,85]. For each year analyzed, individuals that were alive in the community on the first of the year were classified as community members. Differences between individual allele frequencies were assessed using Fisher’s Exact tests, while the differences between total allele frequency distributions were tested using *χ^2^* tests. We used Genepop v4.2 to test for deviations between the observed genotype frequencies, based on the total sampled population of 125 chimpanzees, and the genotype frequencies expected under Hardy-Weinberg equilibrium. An exact Hardy-Weinberg probability was estimated using the Markov chain method [86,87].

### Identification of Fecund Individuals

Both male and female chimpanzees have a social dominance hierarchy in which the dominant individuals produce more offspring. In males, a linear hierarchy results in an alpha male that is dominant over all others, a beta male that is only subordinate to the alpha, and so on [36,75,88,89]. Alpha males sire more offspring (about 30% in the central community) than males of lower rank [36,62,63]. Alpha males also have greater lifetime success, as four of the last seven alpha males were the most successful known males in the central community (S15 Fig). High ranking females also have greater offspring survival and produce offspring faster, and their daughters mature more quickly [64,65]. We therefore categorized fecund individuals in the central community in three ways: 1.) alpha males (N = 7), 2.) fecund females (N = 6), which were those that produced at least twice the mean number of offspring per female, and 3.) fecund females and males combined, where we included both males (N = 2) and females (N = 6) that produced at least twice the mean number of offspring per individual of their sex. Males and females included in the calculation of the mean number of offspring per sex were restricted to those known to have produced at least one offspring since the start of the Gombe study. Both central community males and females produced a mean of three offspring. None of the seven alpha males or six fecund females were infected with SIVcpz. Three were natal females, known to have been born into the community and also to have stayed to reproduce. Two of the other three females were immigrants, and the other was of unknown origins because it was an adult when study of the Gombe chimpanzees began.

Reproductive success was less skewed for females in the northern community. Of the females known to have produced offspring thus far, none have yet produced at least twice the female mean, which was also three. Only three males have been identified as fathers in the northern community thus far, all of which were past or present alpha males and sired four or five offspring each. Therefore, we categorized fecund individuals in the northern community as: 1.) alpha males (N = 3), 2.) females (N = 5) that produced more than the mean number of offspring per female, and 3.) alpha males and fecund females combined. Two of the three alpha males were infected with SIVcpz. None of the four females that were tested were SIVcpz-infected, and none of the five were known to be natal females. Three were identified as immigrants, while the other two were of unknown origins.

## Supporting Information

S1 Data Data for Fig 3. (XLSX)

S2 Data MHC-B sequence alignment for Exon 2 and Exon 3. Included are *MHC-B* allele sequences (Exon 2 and Exon 3) for chimpanzee (*Patr*), human (*HLA*), orangutan (*Popy*), western gorilla (*Gogo*), bonobo (*Papa*), white-handed gibbon (*Hyla*), and African green monkey (*Chsa*). Exon 2 is 270 bases (positions 1–270), and Exon 3 is 276 bases (positions 271–546).
(FAS)

S3 Data Data for Fig 7A. (XLSX)

S4 Data Gombe longitudinal community size and *Patr-B* frequency data. For each allele, the total frequency is given as well as the population frequency contributed by immigrant females who arrived during the study period and their offspring. The contribution of “F lineage” individuals is also given for the central community.
(XLSX)

S5 Data Data for S2 Fig. (XLSX)

S6 Data Data for Fig 10B. (XLSX)

S7 Data Data for Fig 11A. (XLSX)

S8 Data Data for S16 Fig. (XLSX)

S1 Fig Gombe *Patr-B* genotype frequencies and frequency distributions. Every chimpanzee has a *Patr-B* genotype, which is the combination of the two *Patr-B* alleles, one inherited from the mother and the other from the father. Shown here are genotype frequencies and distributions for the total Gombe population (Gombe) and its three constituent social communities (Gombe northern, Gombe central, and Gombe southern). Each of the eleven *Patr-B* alleles is shown in a different colored box. On the left are bar graphs showing the genotypes that are present in each population and their frequencies in descending order. The matrices on the right are divided into two halves by the diagonal from top left to bottom right. The asterisks above the diagonal show all the possible genotypes, whereas the numbers below the diagonal give the observed genotypes and their frequencies as percentages. “Number of individuals” is the population size.
(TIF)

S2 Fig Analysis of neutral genetic markers in Gombe chimpanzees. Shown are the allele frequency distributions for three representative microsatellite loci and the mitochondrial hypervariable D loop in the Gombe population in 2010. The frequency data are provided in S5 Data. The D2S1326 and D2S1333 loci are on chromosome 2, D9S922 is on chromosome 9. Allele N gives the number of alleles for each locus. *P.t.s.* N gives the number of chimpanzees in the 2010 Gombe population. Under “Allele” are given the sizes of the PCR products used to type the various alleles of the microsatellite loci [16,36,40]. For the mitochondrial D loop, the numbers under “Allele” correspond to mtDNA haplotypes reported by Keele et al. 2009 [37]. The frequency-sorted microsatellite and mitochondrial allele distributions significantly differed from the 2010 Gombe distribution of *Patr-B* alleles: (*χ^2^* tests, *p* < 0.001 (D2S1326, D2S1333), *p* < 0.005 (D9S922, hypervariable D loop) (In this analysis only the seven most common alleles were included separately; the frequencies of all other alleles were combined in order to equalize the number of alleles for the various loci).
(TIF)

S3 Fig Phylogeny of *Patr-B* alleles in three subspecies of chimpanzees. (A) Five lineages of chimpanzee *Patr-B* were distinguished by a neighbor-joining phylogenetic tree constructed from the combined nucleotide sequences of exons 2 and 3 (b1-b5, consistent with de Groot et al. 2000 [66]). Alleles associated with a particular chimpanzee subspecies are color-coded: *P. t. schweinfurthii* (green), *P. t. troglodytes* (orange), and *P. t. verus* (blue) (S6 Fig). Underlined alleles are specific to either the Gombe *P. t. schweinfurthii* or BPRC *P. t. verus* population. Alleles with recombinant exons were excluded from the analysis. Nodal bootstrap values are based on 1,000 replications. These values are represented by red circles before nodes when 75%-100%, and light blue squares before nodes when 50%-75% (the full tree is shown in S3B Fig). (B) The complete neighbor-joining tree for *Patr-B* that was simplified to produce S3A Fig. Nodal bootstrap values are based on 1,000 replications. The sequences used to construct these trees are included in S2 Data.
(TIF)

S4 Fig Pairwise comparisons of chimpanzee and human *MHC-B* nucleotide diversity. (A-O) The pairwise nucleotide differences (p-distances) for each *MHC-B* allele set (exons 2 and 3) are plotted as histograms (as summarized in Fig 4). “*P.t.s.*” is *P. t. schweinfurthii*; “*P.t.t.*” is *P. t. troglodytes*; “*P.t.v.*” is *P. t. verus*. Between-group comparisons include only pairs of alleles from different groups. M: mean p-distance; N: number of alleles. (P) Statistical results of the unpaired *t* tests between the mean p-distances for different *MHC-B* allele pair sets from Fig 4 and S4A-S4O Fig *p*-values are given when significant, while NS indicates no significance. Gray indicates that a particular combination was not tested. The alleles used to generate the p-distances are listed in S6 Fig, and their sequences are included in S2 Data.
(TIF)

S5 Fig Substitutions distinguishing novel Gombe *Patr-B* alleles and allotypes. Seven of eleven Gombe *Patr-B* represented new discoveries and are shown here. Their sequences are provided in S2 Data. “Closest allele” gives the most similar *Patr-B* allele for each exon found in the literature according to nucleotide sequence. Two alternative possibilities are given for Patr-B*39:01. Alleles with identical exon sequences are separated by a backslash “/.” Under “Residue” are given the numbers of the codons (which correspond to the amino acid sequence) containing the nucleotide substitutions. Out of a total of 16 nucleotide substitutions, 15 of them (in bold) are nonsynonymous changes. This strong bias towards substitutions that alter the structure of the Patr-B protein reflects the role of natural selection in generating and maintaining *Patr-B* polymorphism.
(TIF)

S6 Fig *MHC-B* alleles used in the phylogenetic analyses. *Patr-B* alleles were all those identified from three chimpanzee subspecies (*P. t. schweinfurthii* [*P.t.s.*], *P. t. troglodytes* [*P.t.t.*], and *P. t. verus* [*P.t.v.*]). Other *MHC-B* were from orangutan (*Popy-B*), western gorilla (*Gogo-B*), bonobo (*Papa-B*), and human (*HLA-B*). The *MHC-B* core alleles for each species were selected to represent the scope and breadth of the polymorphism (Fig 1). The 11 Gombe *P. t. schweinfurthii* alleles and 10 BPRC *P. t. verus* alleles are highlighted in gray. The seven novel Gombe *P. t. schweinfurthii* alleles are in bold. All sequences listed are included in S2 Data.
(TIF)

S7 Fig Hominoid *MHC-B* exon 2 phylogeny. Complete neighbor-joining trees corresponding to the simpler trees shown in Fig 8. (A) A tree is constructed from the nucleotide sequences of exon 2 for representative *MHC-B* alleles and including Patr-B*06:03 and three other Gombe *Patr-B* alleles (in bold blue). African green monkey (*Chlorocebus sabaeus*) *MHC-B* was used as the outgroup (*Chsa*). This tree shows that Patr-B*06:03 groups with HLA-B*57:01 in a unique trans-species *MHC-B* clade (gray box). This unique clade is maintained in trees constructed from the sequence of codons 62–74 (B) but not in trees constructed from exon 2 sequences in which codons 62–74 were deleted (C). Nodal bootstrap values are based on 1,000 replications. Species designations of *MHC-B*: chimpanzee (*Patr*), bonobo (*Papa*), human (*HLA*), orangutan (*Popy*), western gorilla (*Gogo*), and white-handed gibbon (*Hyla*). All sequences used to generate the trees are included in S2 Data.
(TIF)

S8 Fig The success rate for SIVcpz vRNA amplification from fecal samples does not differ between the three Gombe chimpanzee communities. For SIVcpz-infected individuals, not all fecal samples gave PCR amplification of vRNA. The number of samples giving successful amplification correlated with the total number of samples tested. Similar correlations, as seen from the trend lines and correlation coefficients (R) were observed for the northern (A), central (B), and southern (C) communities, as well as the total Gombe population (D). N represents the number of fecal samples tested, followed in parentheses by the percentage of samples giving successful amplification. Black diamonds represent single individuals, whereas gray diamonds indicate two or more individuals having the same sampling ratio. The data used to generate this figure are given in S2 Table.
(TIF)

S9 Fig The success rate of SIVcpz vRNA amplification from feces of infected chimpanzees varies with Patr-B type. Data are represented as both histograms (A-D) and scatterplots (E-I). (A-B) The range and distribution of the total number of fecal samples tested per individual is similar for Patr-B*06:03 positive (A) and Patr-B*06:03-negative (B) chimpanzees. (C-D) Overall, a smaller proportion of fecal samples from Patr-B*06:03-positive chimpanzees yielded vRNA amplification (C), compared to the fecal samples from Patr-B*06:03-negative chimpanzees (D). The data used to generate this figure are given in S2 Table. The correlation between the number of fecal samples tested and the number giving successful SIVcpz vRNA amplification is shown in (E) for all SIVcpz-infected chimpanzees. This is compared to individuals who have Patr-B*06:03 (F) or lack Patr-B*06:03 (G). vRNA is more frequently amplified from the samples from individuals who lack B*06:03 than from the individuals who have B*06:03, consistent with B*06:03 having the effect of reducing VL. Similar comparison for Patr-B*39:01 (H and I) shows that this Patr-B allotype correlates with increased frequency of vRNA amplification. This is consistent with Patr-B*39:01 increasing the VL (S10C Fig). N represents the number of fecal samples tested. Black diamonds represent single individuals, whereas gray diamonds indicate two or more individuals having the same sampling ratio. In each panel the trend line is plotted and the correlation coefficient (R) is given.
(TIF)

S10 Fig Modeling the effects of community and *Patr-B* type on the rates of SIVcpz vRNA amplification and infected chimpanzee survival. In this context, the rate is the proportion of fecal samples that give successful amplification of SIVcpz vRNA. Statistics include the estimate, which gives the magnitude and direction of the effect for each variable, and the 95% confidence intervals (95% CI). The intercept is the predicted mean value of the dependent variable (e.g., amplification rate) when all the independent variables are set to zero. (A) Rates of vRNA amplification did not vary significantly according to the community in which the samples were collected. (B) When the Patr-B*06:03, B*22:04, and B*39:01 allotypes were tested in combination, only Patr-B*06:03 was associated with a significantly lower rate of vRNA amplification. This result suggests that Patr-B*06:03 has the effect of reducing the VL. (C) When the Patr-B*06:03, B*22:04 and B*39:01 allotypes were tested individually, B*06:03 was again correlated significantly with a lower rate of amplification, whereas B*22:04 and B*39:01 were associated with higher amplification rates. Thus, it is possible that Patr-B*22:04 and Patr-B*39:01 are causing increases in the VL. However, the effect for Patr-B*22:04 is negative (indicative of lower VL) when tested in combination (B). (D) None of the six *Patr-B* allotypes examined were correlated with either increased or decreased survival of SIVcpz-infected chimpanzees. Applying either the more conservative or less conservative criteria (described in Materials and Methods and S1 Text) gave the same results. Values for Patr-B*39:01 were not reported for the more conservative analysis because only three of the chimpanzees included in the analysis have B*39:01, and by the criteria used in the analysis none of these were categorized as dead. Consequently, the estimate and 95% CI were extraordinarily large and not informative.
(TIF)

S11 Fig Variability of four HLA-B*57 HIV Gag epitopes as found in SIVcpz Gag. SIVcpz Gag protein sequences were obtained from the HIV sequence database (http://www.hiv.lanl.gov/) and aligned against the four HLA-B*57 restricted HIV-1 Gag epitopes. “*P.t.s.*” identifies Gag sequences from *P. t. schweinfurthii*, while “*P.t.t.*” identifies sequences from *P. t. troglodytes*. Two epitopes predominantly have serine (S) at the P2 peptide anchor position (in gray). Position 242 (in black) is the site of the T242N HIV-1 viral escape mutation that negatively impacts viral replication potential and frequently occurs in individuals with HLA-B*57 [58,90]. SIVcpz Gag sequences present in the Gombe population are in bold and have an associated, chimpanzee-specific CH identification number (which corresponds to the chimpanzee ID number given in S1 Table). **The individual from which the viral sequence was obtained has Patr-B*06:03 or has Patr-B*22:05. Location of infected animals: Tanzania (TZ), Democratic Republic of the Congo (DRC), Cameroon (CM), Gabon (GA), and the United States (US).
(TIF)

S12 Fig Temporal variation of Gombe community frequency-sorted *Patr-B* allele distributions. (A) *Patr-B* N is the number of alleles, while *P.t.s* N is the number of individuals genotyped for *Patr-B* out of the total number of individuals alive on the first day of the year. Black brackets indicate significant differences in allele frequencies between the three most frequent alleles within a community at a census time: narrow brackets (south: 1998, north: 2000 and 2005) indicate a difference in the frequencies between the two most frequent alleles; wide brackets in the central community (all years) indicate that the two most frequent alleles are at a significantly higher frequency than the third allele; the bracket between alleles in the southern community in 2000 shows that the highest frequency allele is significantly elevated compared to the third highest frequency allele (Fisher’s Exact tests, *p*-values given below the brackets (*p* < 0.0001 applies to both high frequency alleles in the central community)). (B) *χ^2^* test statistics of differences between the total allele frequency distributions within community (compared to the previous year) and between community (within the same year). In this analysis, only the three most common alleles were included individually; all other alleles were combined so as to equalize the number of alleles in the northern (N), central (C), and southern (S) communities. Bold highlights significant results (*χ^2^*, significant *p*-value). Statistics for 2010 first gives the comparison with 2005, and second, the comparison with the earliest time point available for the community (1995 (N and C) or 1998 (S)). Frequencies used in this figure are provided in S4 Data.
(TIF)

S13 Fig Temporal variation and the contribution of immigrant females to the *Patr-B* allele distribution in the three Gombe communities. Gray proportions of bars represent the contribution of immigrant females that arrived during the study period and their offspring. *Patr-B* N represents the number of alleles present within the community. *P.t.s* N represents the number of individuals that were genotyped for *Patr-B* out of the total number of individuals alive on the first of the year. Asterisks denote allele frequency differences between communities within the same year (north (N); central (C)) (Fisher’s Exact tests, *p*-values given below). There were no differences between 1995 (or 1998 for the southern community) and 2010 when comparing within the communities. The data for the northern and southern communities are also presented in Fig 9B and 9C. Frequencies used in this figure are given in S4 Data.
(TIF)

S14 Fig Gombe female immigrants between 1990 and 2010. Identification numbers distinguish the females. Females are grouped according to the community “From” which they departed, the community “To” which they went, and then by the date of transfer (“Start Date”, month and year) (unknown (U); north (N, blue); central (C, yellow); south (S, purple). An underlined “S” in the “From” column indicates that the female was inferred to have emigrated from the southern community. Due to the lack of habituation of the southern community, it is possible that some females appearing in the community from unknown origins were actually natal females. Asterisks indicate that the female reproduced within a community. “End Date” lists when the female departed for another community or is null if she still resides there. Two females (CH093 and CH096) had more than one emigration. Females that died (D) or are suspected to have died (U/D) are noted under “Dead.” SIVcpz-infected chimpanzees have “Yes” under “SIV.” The *Patr-B* genotype for each female is also provided. The females are further grouped according to the *Patr-B* census years between which their community transfers occurred.
(TIF)

S15 Fig 1984–2010 approximate timeline for alpha males in the Gombe central community. Each timeline increment represents one year. The approximate start and end years of alpha males’ tenures are labeled, and tenure periods alternate between light and dark gray. ID numbers distinguish the alpha males. CH010 was still the alpha male as of the end of 2014. Asterisks identify the F matriline males. The number of offspring sired by each male while occupying the alpha position is given first followed by the total offspring sired (currently known, or lifetime) in parentheses [35,36,79]. The top row of numbers is for all known offspring, while the bottom row is for offspring that were *Patr-B* genotyped in this study. The star marks the year (2000) in which noninvasive fecal sampling into RNAlater began to obtain SIVcpz vRNA and DNA suitable for sequencing.
(TIF)

S16 Fig *Patr-B* frequency distributions for the offspring of more-fecund and less-fecund individuals. *Patr-B* N represents the number of alleles present within the offspring produced, while *P.t.s* N represents the number of offspring produced. Central community offspring with known fathers were sired by seven fecund (alpha) males, and six less fecund males sired the remaining offspring. Central community offspring with known mothers were produced by six fecund females and 23 less fecund females. “F Matriline” represents offspring produced by males and females from three generations of the “F” maternal lineage in the central community. Identified parents of northern community offspring included three fecund (alpha) males, five fecund females, and nine less-fecund females. The frequency distributions of offspring produced by females in the central community were significantly different ([**], *χ^2^* = 8.202, *p* < 0.05). In this analysis, only the three most common alleles were included individually; all other alleles were combined so as to equalize the number of alleles between the two groups. Differences in individual allele frequencies are noted by giving *p*-values when they were significantly different between fecund and less fecund distributions, and also ** when they were different than that of the 2010 distribution (Fisher’s Exact tests). A (+) indicates a higher frequency, while (-) indicates a lower frequency. Frequencies used in this figure are provided in S8 Data.
(TIF)

S1 Table *Patr-B* genotypes of 125 Gombe chimpanzees. Each chimpanzee has a unique identification number, except “AND,” a chimpanzee named Andromeda who died at a young age, before a fecal sample could be obtained and an identification number assigned. For males, the identification numbers are underlined, for females they are not. In the column headed “SIV,” the word “Yes” means the individual is infected with SIVcpz. In the column headed “Sample,” the identification number of a fecal sample or the word “tissue” indicating an organ or tissue collected during necropsy. An asterisk denotes samples that were not genotyped for microsatellite markers. Presence of “(2)” or “(3)” next to a sample name or number indicates that two or three independent PCR were performed on the sample. The columns headed “*Patr-B*” give the two *Patr-B* alleles for each chimpanzee; they can be different (heterozygous) or identical (homozygous). The names of novel alleles discovered in this study are in bold. Each box is colored according to the *Patr-B* allele it contains. The four central columns give the exon 2 and 3 typing of each fecal sample. In the columns headed “Clones,” “DS” denotes that an allele was defined by direct sequencing (DS). A number in these columns shows the allele was defined by cloning and sequencing, and gives the number of clones sequenced. A blank box in these columns indicates that a single form of exon 2 or 3 was detected, because the individual is homozygous for that exon. The columns headed “Pedigree” at the right identify a chimpanzee’s mother (under “Maternal”) and father (under “Paternal”), where known.
(XLSX)

S2 Table Characteristics of the chimpanzee fecal samples used for the detection SIVcpz vRNA. Listed are samples of chimpanzee feces that were all shown to contain SIVcpz-specific antibodies by western blotting. These samples are grouped according to the 30 chimpanzees from which they were collected. For each chimpanzee, the identity numbers and the *Patr-B* types are given. For each fecal sample, the identity number, the date of collection, and the community territory (northern [N], central [C], or southern [S]) in which the collection was made, are given. These data are followed by the results of testing for the presence (+) or absence (-) of vRNA detected by PCR amplification. For each individual, the total number of fecal samples tested is given, as well as the number shown to contain vRNA. For chimpanzees from which vRNA was detected, the infecting strain of SIVcpz is identified by TAN followed by a number from 1 to 23.
(XLS)

S1 Text Supplemental materials and methods. (DOCX)

## Abbreviations

AIDS: acquired immune deficiency syndrome
BPRC: Biomedical Primate Research Center
HIV: human immunodeficiency virus
KIR: Killer cell Immunoglobulin-like Receptor
MHC: major histocompatibility complex
NK: natural killer
SIVcpz: simian immunodeficiency virus from chimpanzees
VL: viral load
vRNA: viral RNA
