# Supplementary figures and images for: Signature Patterns of MHC Diversity in Three Gombe Communities of Wild Chimpanzees Reflect Fitness in Reproduction and Immune Defense against SIVcpz
Source: PLoS Biol. 2015 May 28;13(5):e1002144. doi: 10.1371/journal.pbio.1002144 (PMC4447270; doi:10.1371/journal.pbio.1002144)

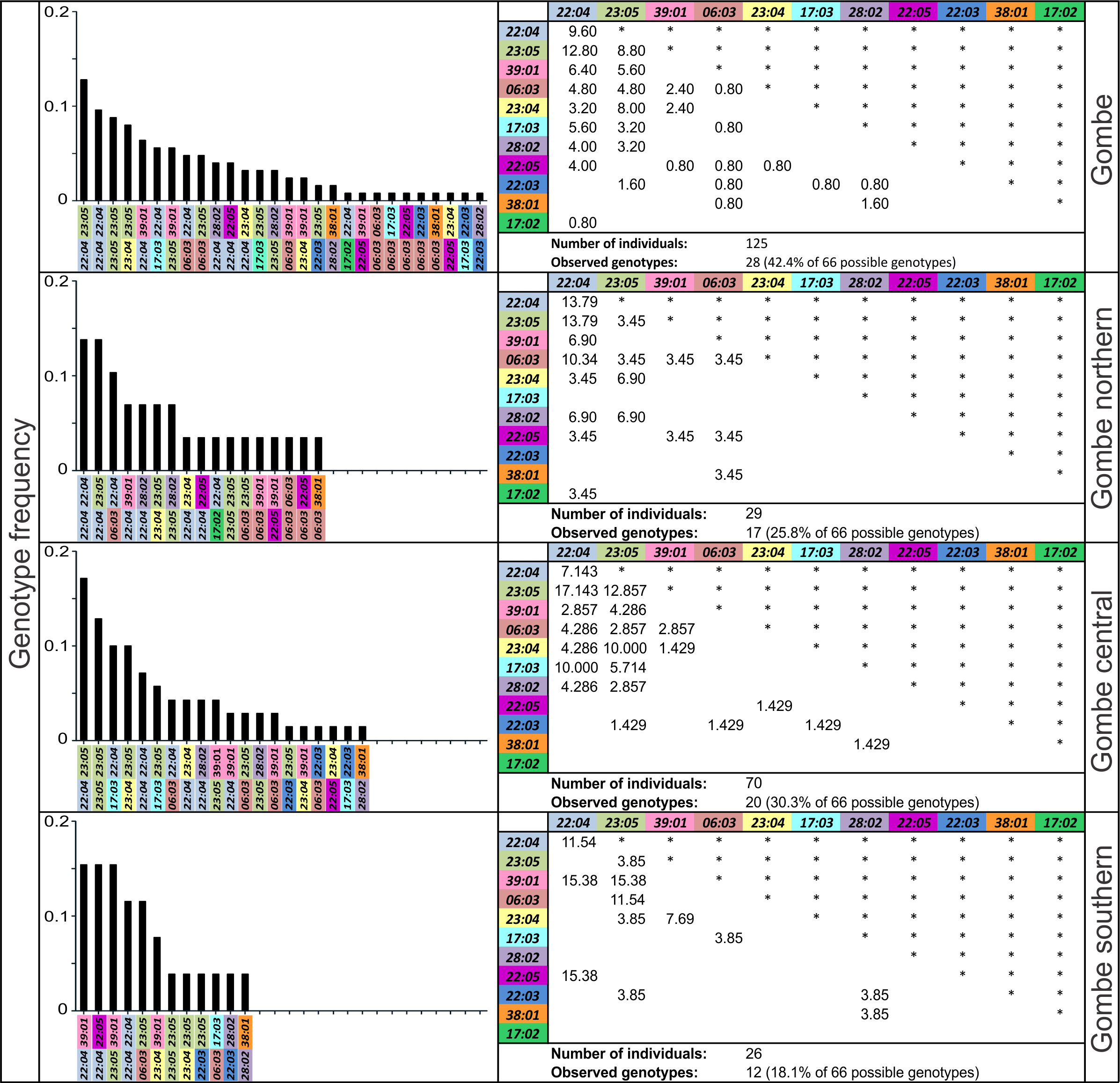

Supplement: S1 Fig — Every chimpanzee has a Patr-B genotype, which is the combination of the two Patr-B alleles, one inherited from the mother and the other from the father. Shown here are genotype frequencies and distributions for the total Gombe population (Gombe) and its three constituent social communities (Gombe northern, Gombe central, and Gombe southern). Each of the eleven Patr-B alleles is shown in a different colored box. On the left are bar graphs showing the genotypes that are present in each population and their frequencies in descending order. The matrices on the right are divided into two halves by the diagonal from top left to bottom right. The asterisks above the diagonal show all the possible genotypes, whereas the numbers below the diagonal give the observed genotypes and their frequencies as percentages. “Number of individuals” is the population size. (TIF) [file pbio.1002144.s009.tif]

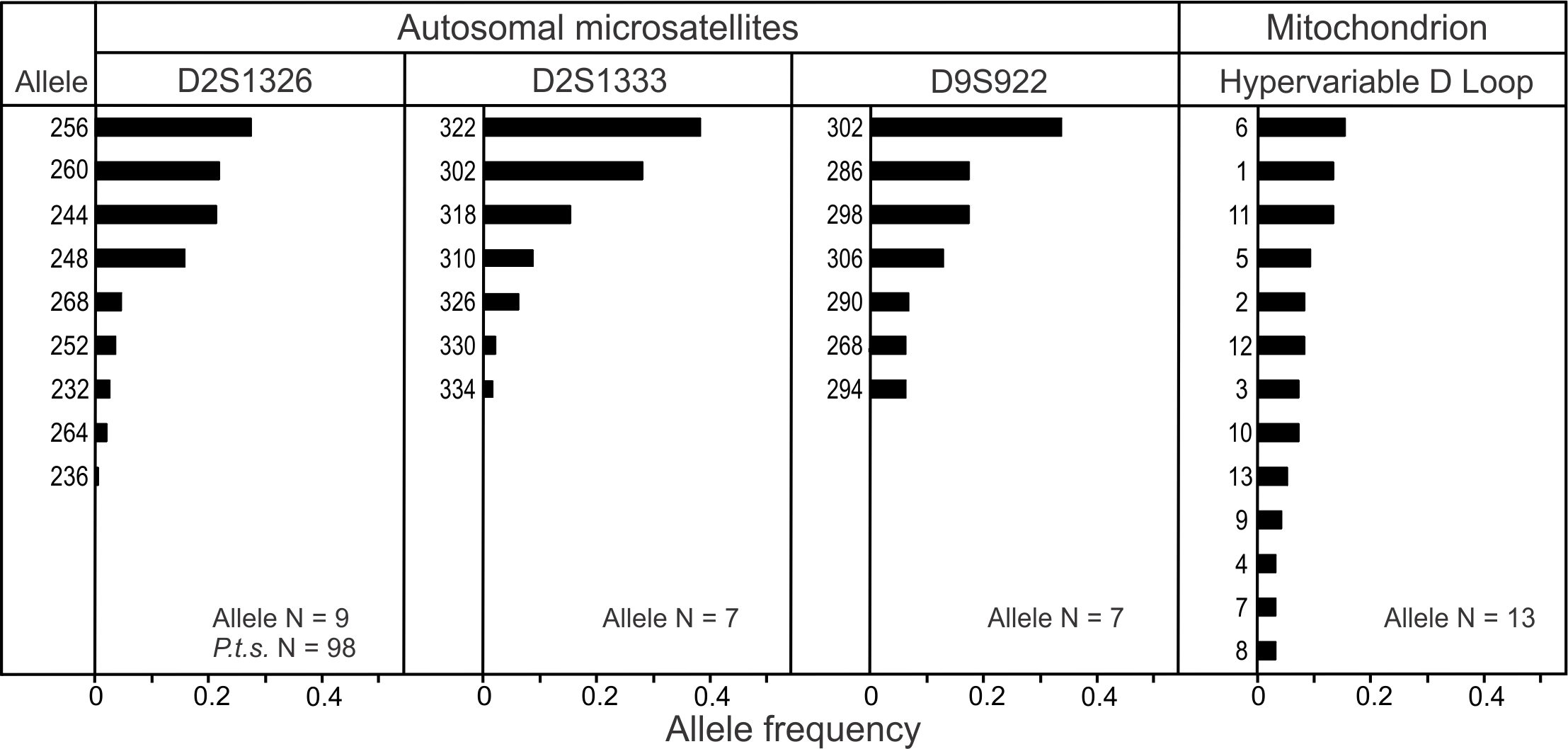

Supplement: S2 Fig — Shown are the allele frequency distributions for three representative microsatellite loci and the mitochondrial hypervariable D loop in the Gombe population in 2010. The frequency data are provided in S5 Data. The D2S1326 and D2S1333 loci are on chromosome 2, D9S922 is on chromosome 9. Allele N gives the number of alleles for each locus. P.t.s. N gives the number of chimpanzees in the 2010 Gombe population. Under “Allele” are given the sizes of the PCR products used to type the various alleles of the microsatellite loci [16,36,40]. For the mitochondrial D loop, the numbers under “Allele” correspond to mtDNA haplotypes reported by Keele et al. 2009 [37]. The frequency-sorted microsatellite and mitochondrial allele distributions significantly differed from the 2010 Gombe distribution of Patr-B alleles: (χ2 tests, p < 0.001 (D2S1326, D2S1333), p < 0.005 (D9S922, hypervariable D loop) (In this analysis only the seven most common alleles were included separately; the frequencies of all other alleles were combined in order to equalize the number of alleles for the various loci). (TIF) [file pbio.1002144.s010.tif]

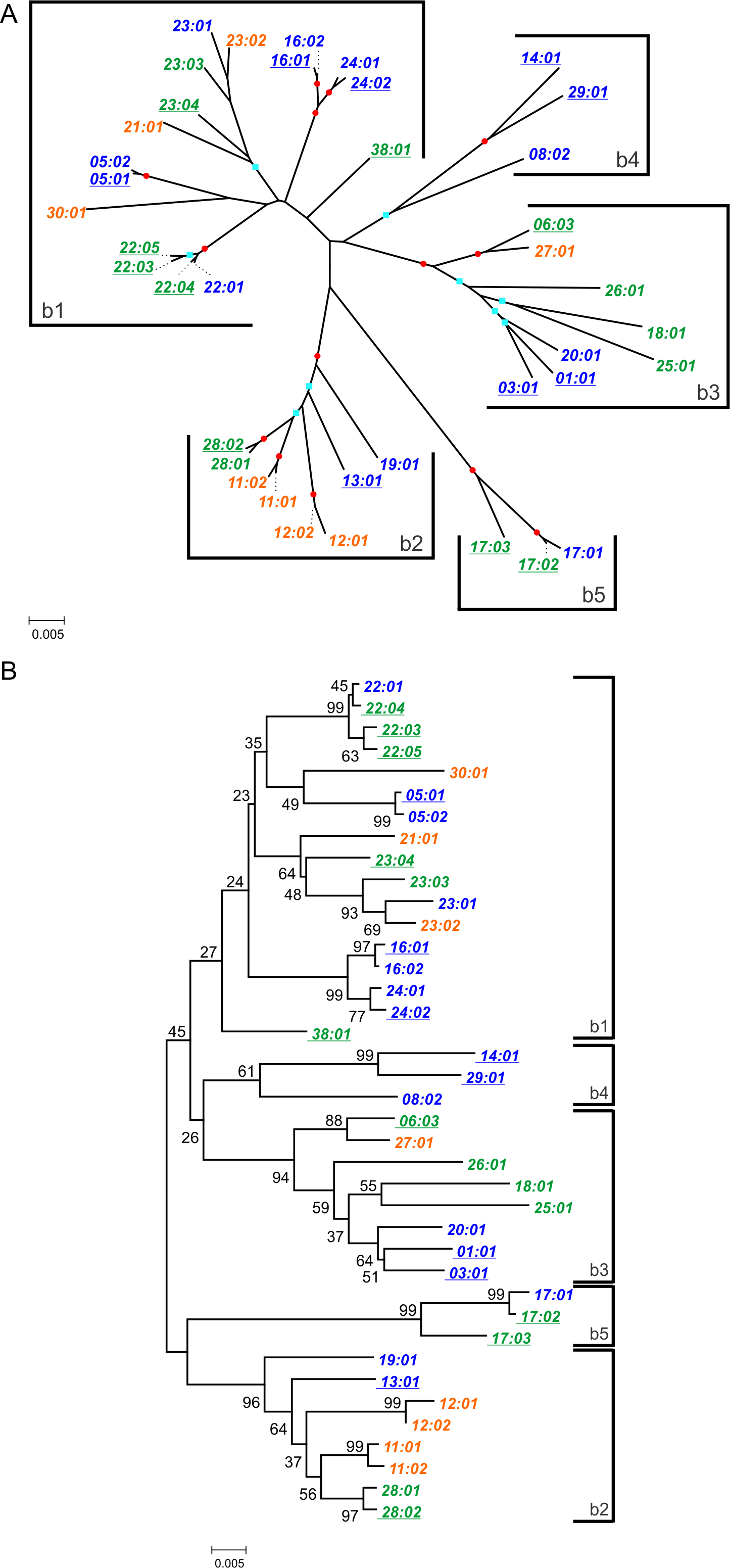

Supplement: S3 Fig — (A) Five lineages of chimpanzee Patr-B were distinguished by a neighbor-joining phylogenetic tree constructed from the combined nucleotide sequences of exons 2 and 3 (b1-b5, consistent with de Groot et al. 2000 [66]). Alleles associated with a particular chimpanzee subspecies are color-coded: P. t. schweinfurthii (green), P. t. troglodytes (orange), and P. t. verus (blue) (S6 Fig). Underlined alleles are specific to either the Gombe P. t. schweinfurthii or BPRC P. t. verus population. Alleles with recombinant exons were excluded from the analysis. Nodal bootstrap values are based on 1,000 replications. These values are represented by red circles before nodes when 75%-100%, and light blue squares before nodes when 50%-75% (the full tree is shown in S3B Fig). (B) The complete neighbor-joining tree for Patr-B that was simplified to produce S3A Fig. Nodal bootstrap values are based on 1,000 replications. The sequences used to construct these trees are included in S2 Data. (TIF) [file pbio.1002144.s011.tif]

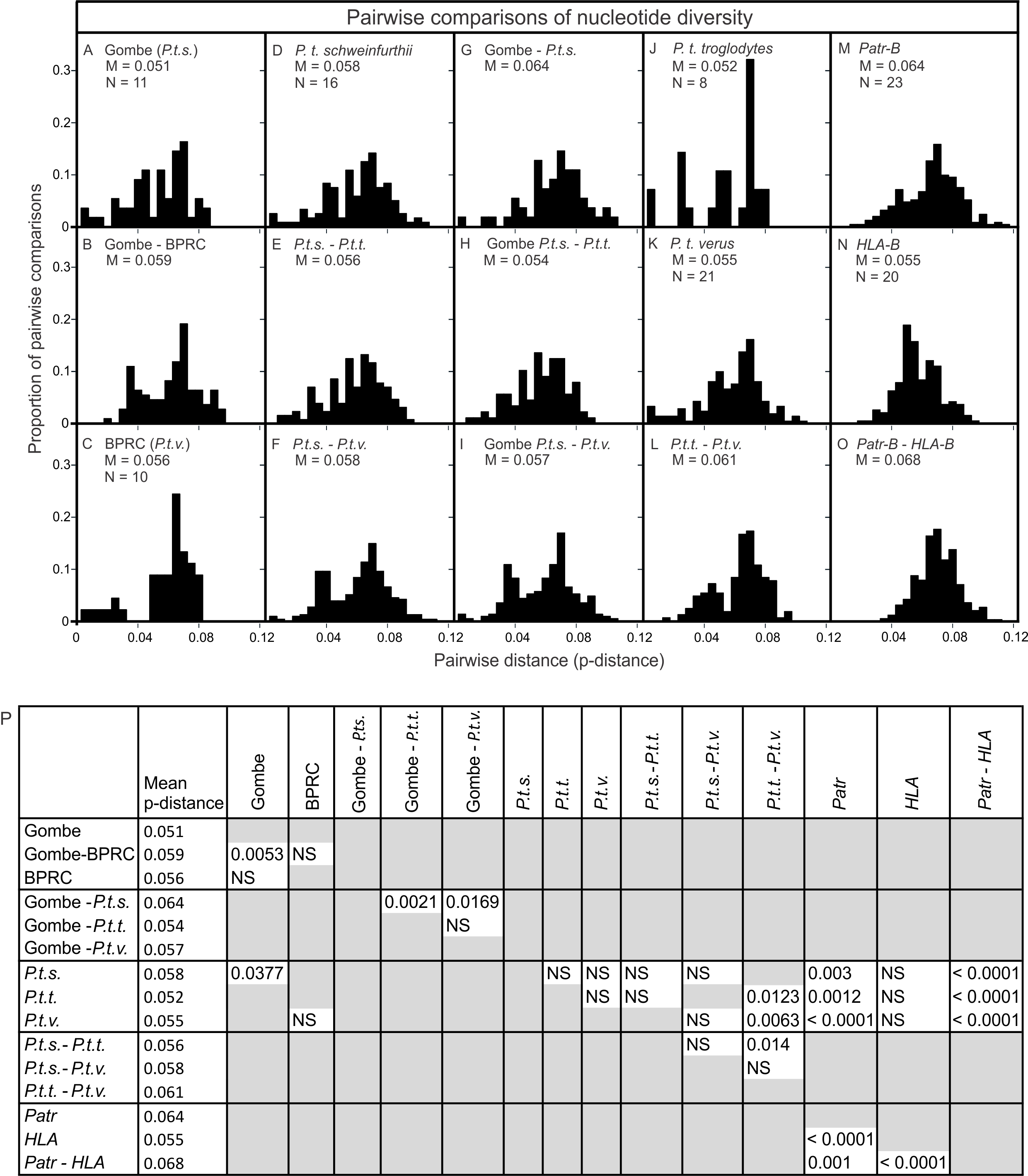

Supplement: S4 Fig — (A-O) The pairwise nucleotide differences (p-distances) for each MHC-B allele set (exons 2 and 3) are plotted as histograms (as summarized in Fig 4). “P.t.s.” is P. t. schweinfurthii; “P.t.t.” is P. t. troglodytes; “P.t.v.” is P. t. verus. Between-group comparisons include only pairs of alleles from different groups. M: mean p-distance; N: number of alleles. (P) Statistical results of the unpaired t tests between the mean p-distances for different MHC-B allele pair sets from Fig 4 and S4A-S4O Fig p-values are given when significant, while NS indicates no significance. Gray indicates that a particular combination was not tested. The alleles used to generate the p-distances are listed in S6 Fig, and their sequences are included in S2 Data. (TIF) [file pbio.1002144.s012.tif]

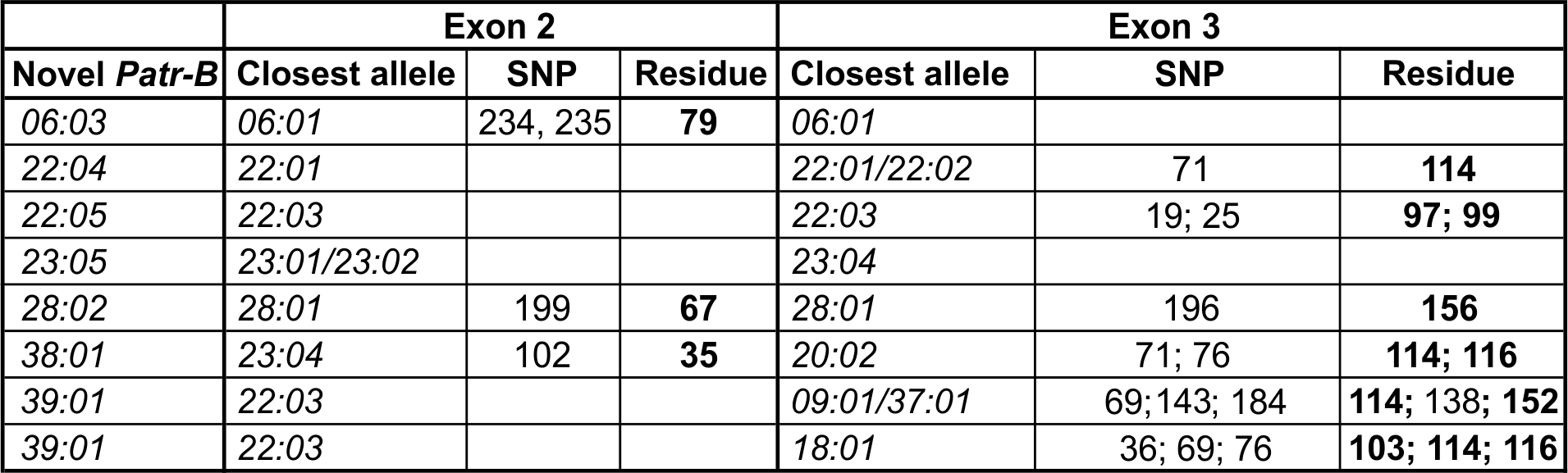

Supplement: S5 Fig — Seven of eleven Gombe Patr-B represented new discoveries and are shown here. Their sequences are provided in S2 Data. “Closest allele” gives the most similar Patr-B allele for each exon found in the literature according to nucleotide sequence. Two alternative possibilities are given for Patr-B*39:01. Alleles with identical exon sequences are separated by a backslash “/.” Under “Residue” are given the numbers of the codons (which correspond to the amino acid sequence) containing the nucleotide substitutions. Out of a total of 16 nucleotide substitutions, 15 of them (in bold) are nonsynonymous changes. This strong bias towards substitutions that alter the structure of the Patr-B protein reflects the role of natural selection in generating and maintaining Patr-B polymorphism. (TIF) [file pbio.1002144.s013.tif]

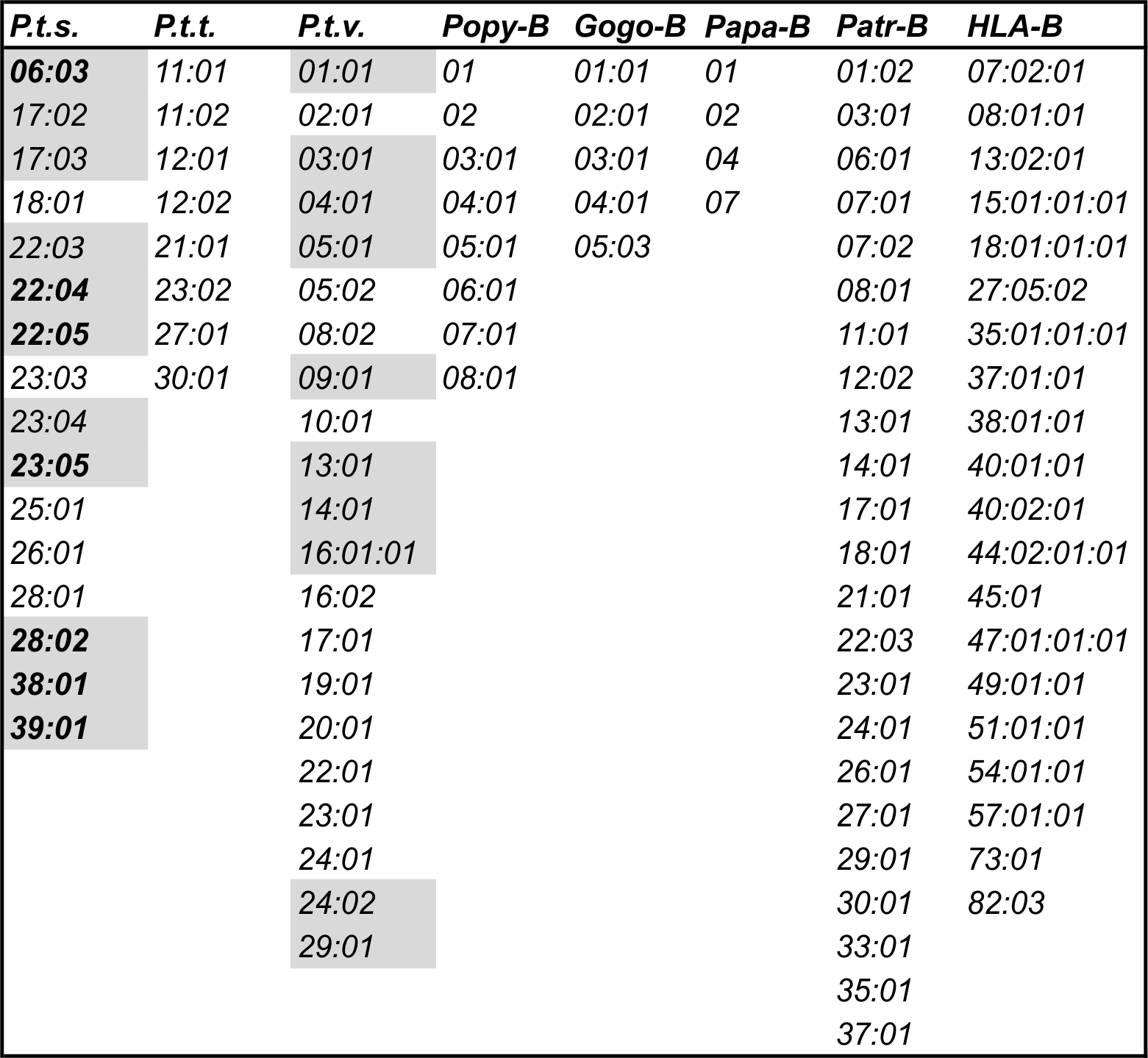

Supplement: S6 Fig — Patr-B alleles were all those identified from three chimpanzee subspecies (P. t. schweinfurthii [P.t.s.], P. t. troglodytes [P.t.t.], and P. t. verus [P.t.v.]). Other MHC-B were from orangutan (Popy-B), western gorilla (Gogo-B), bonobo (Papa-B), and human (HLA-B). The MHC-B core alleles for each species were selected to represent the scope and breadth of the polymorphism (Fig 1). The 11 Gombe P. t. schweinfurthii alleles and 10 BPRC P. t. verus alleles are highlighted in gray. The seven novel Gombe P. t. schweinfurthii alleles are in bold. All sequences listed are included in S2 Data. (TIF) [file pbio.1002144.s014.tif]

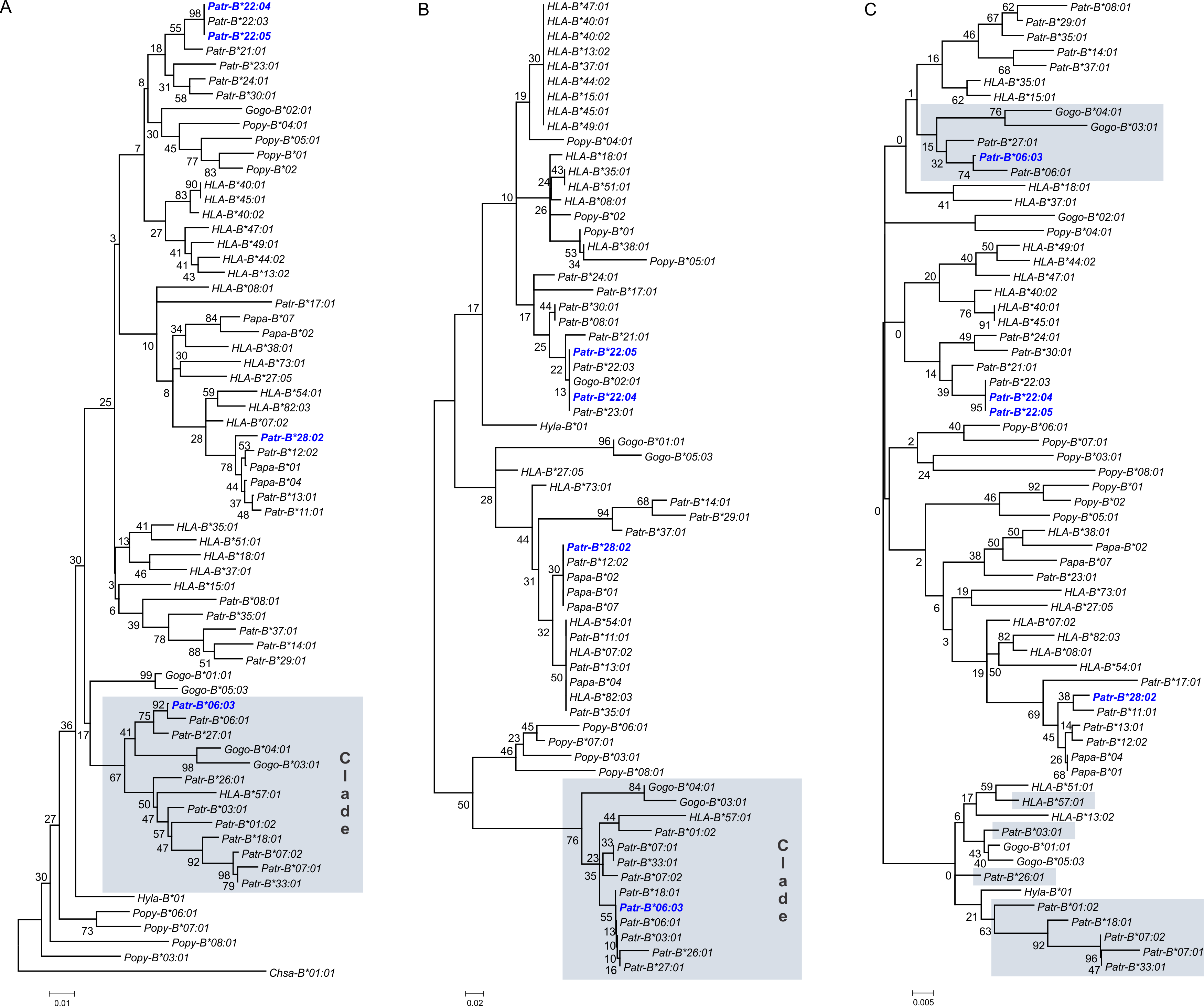

Supplement: S7 Fig — Complete neighbor-joining trees corresponding to the simpler trees shown in Fig 8. (A) A tree is constructed from the nucleotide sequences of exon 2 for representative MHC-B alleles and including Patr-B*06:03 and three other Gombe Patr-B alleles (in bold blue). African green monkey (Chlorocebus sabaeus) MHC-B was used as the outgroup (Chsa). This tree shows that Patr-B*06:03 groups with HLA-B*57:01 in a unique trans-species MHC-B clade (gray box). This unique clade is maintained in trees constructed from the sequence of codons 62–74 (B) but not in trees constructed from exon 2 sequences in which codons 62–74 were deleted (C). Nodal bootstrap values are based on 1,000 replications. Species designations of MHC-B: chimpanzee (Patr), bonobo (Papa), human (HLA), orangutan (Popy), western gorilla (Gogo), and white-handed gibbon (Hyla). All sequences used to generate the trees are included in S2 Data. (TIF) [file pbio.1002144.s015.tif]

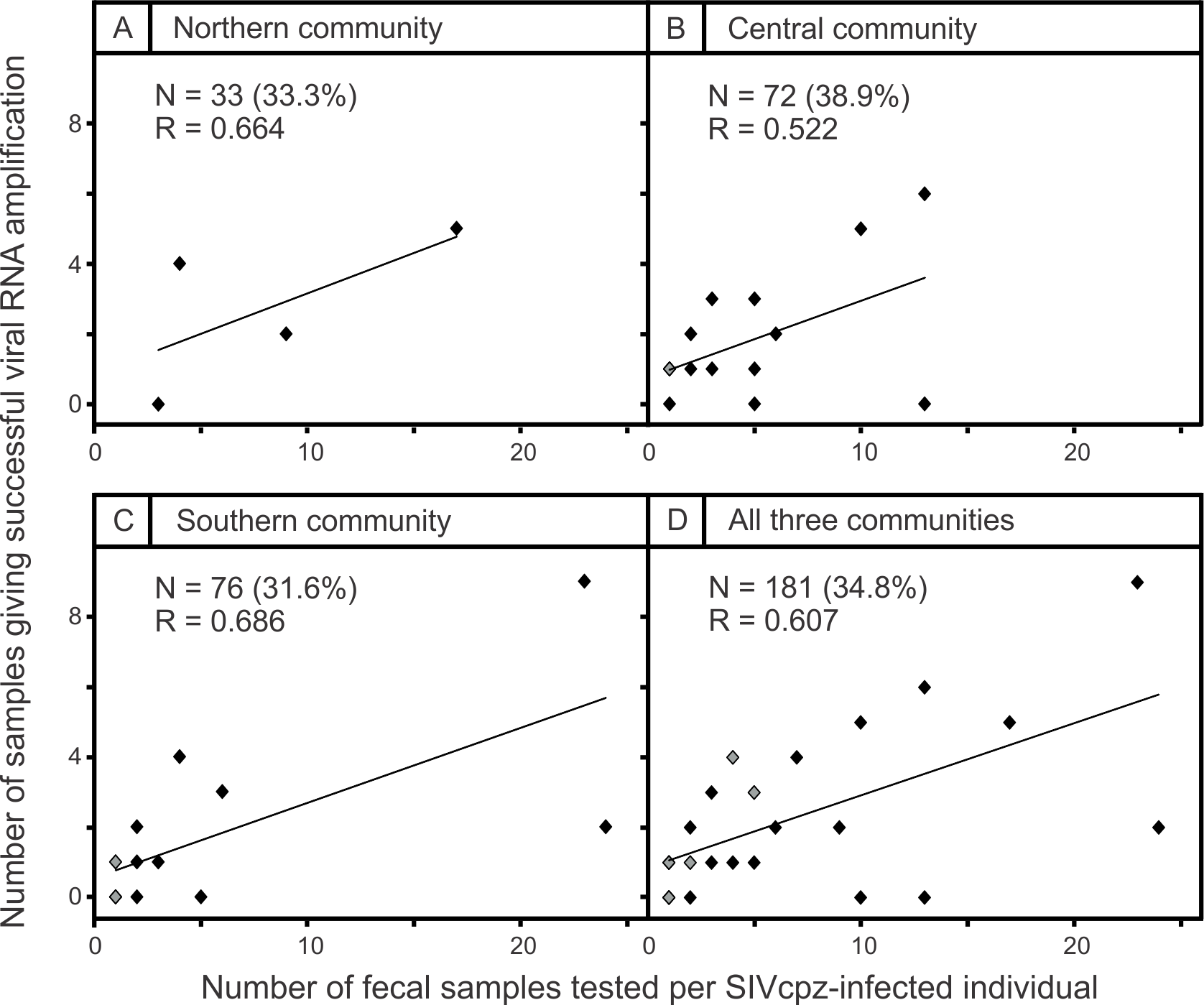

Supplement: S8 Fig — For SIVcpz-infected individuals, not all fecal samples gave PCR amplification of vRNA. The number of samples giving successful amplification correlated with the total number of samples tested. Similar correlations, as seen from the trend lines and correlation coefficients (R) were observed for the northern (A), central (B), and southern (C) communities, as well as the total Gombe population (D). N represents the number of fecal samples tested, followed in parentheses by the percentage of samples giving successful amplification. Black diamonds represent single individuals, whereas gray diamonds indicate two or more individuals having the same sampling ratio. The data used to generate this figure are given in S2 Table. (TIF) [file pbio.1002144.s016.tif]

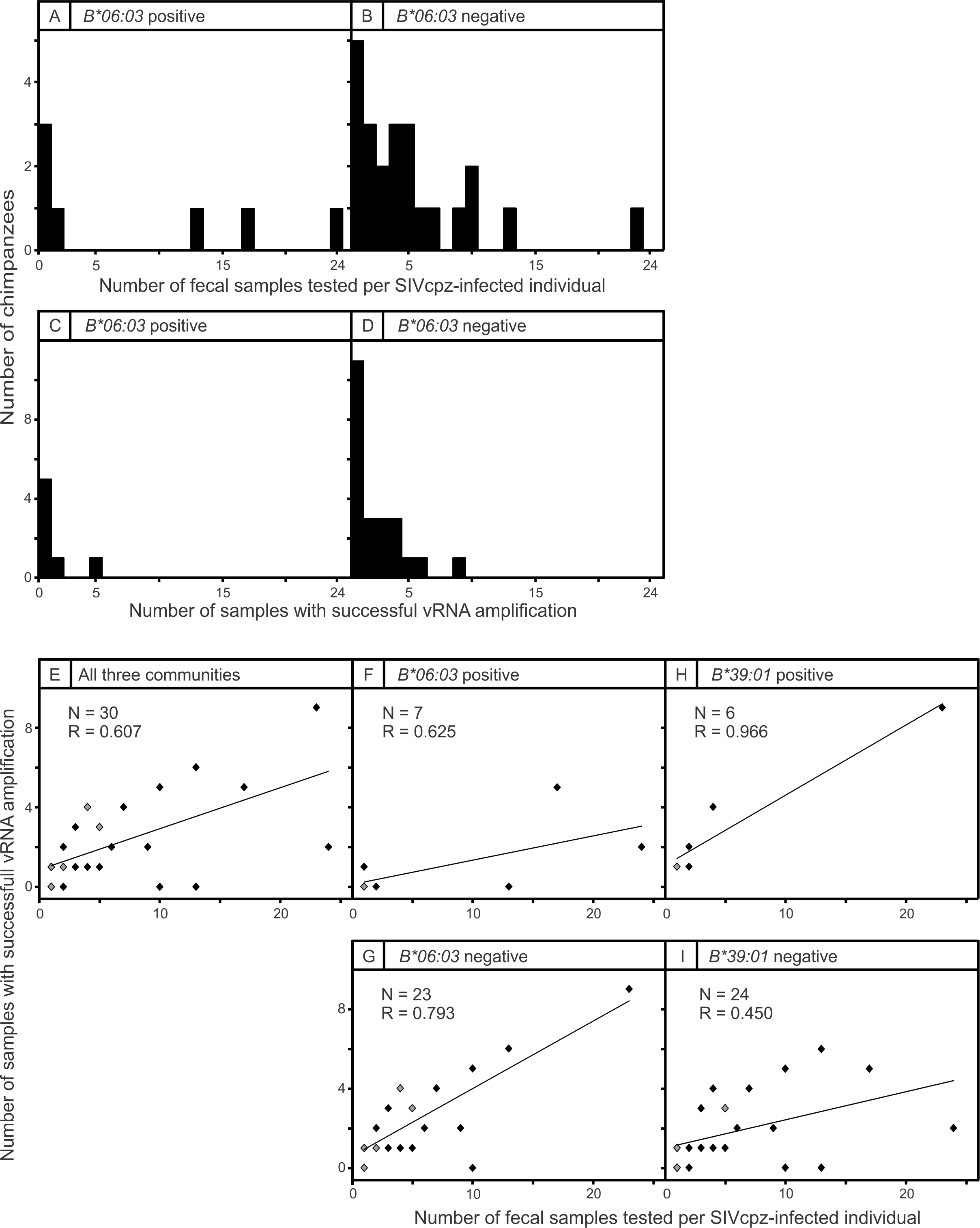

Supplement: S9 Fig — Data are represented as both histograms (A-D) and scatterplots (E-I). (A-B) The range and distribution of the total number of fecal samples tested per individual is similar for Patr-B*06:03 positive (A) and Patr-B*06:03-negative (B) chimpanzees. (C-D) Overall, a smaller proportion of fecal samples from Patr-B*06:03-positive chimpanzees yielded vRNA amplification (C), compared to the fecal samples from Patr-B*06:03-negative chimpanzees (D). The data used to generate this figure are given in S2 Table. The correlation between the number of fecal samples tested and the number giving successful SIVcpz vRNA amplification is shown in (E) for all SIVcpz-infected chimpanzees. This is compared to individuals who have Patr-B*06:03 (F) or lack Patr-B*06:03 (G). vRNA is more frequently amplified from the samples from individuals who lack B*06:03 than from the individuals who have B*06:03, consistent with B*06:03 having the effect of reducing VL. Similar comparison for Patr-B*39:01 (H and I) shows that this Patr-B allotype correlates with increased frequency of vRNA amplification. This is consistent with Patr-B*39:01 increasing the VL (S10C Fig). N represents the number of fecal samples tested. Black diamonds represent single individuals, whereas gray diamonds indicate two or more individuals having the same sampling ratio. In each panel the trend line is plotted and the correlation coefficient (R) is given. (TIF) [file pbio.1002144.s017.tif]

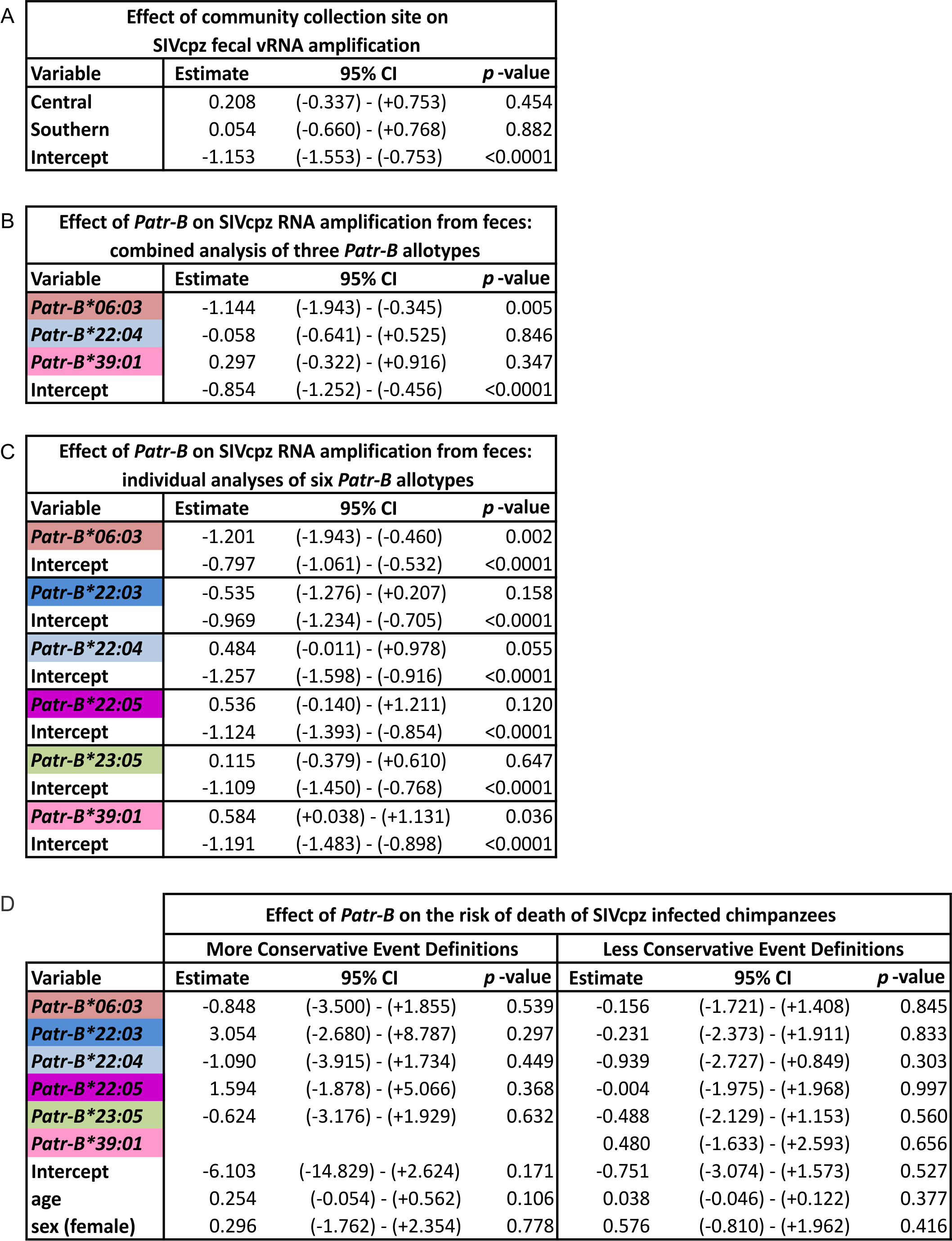

Supplement: S10 Fig — In this context, the rate is the proportion of fecal samples that give successful amplification of SIVcpz vRNA. Statistics include the estimate, which gives the magnitude and direction of the effect for each variable, and the 95% confidence intervals (95% CI). The intercept is the predicted mean value of the dependent variable (e.g., amplification rate) when all the independent variables are set to zero. (A) Rates of vRNA amplification did not vary significantly according to the community in which the samples were collected. (B) When the Patr-B*06:03, B*22:04, and B*39:01 allotypes were tested in combination, only Patr-B*06:03 was associated with a significantly lower rate of vRNA amplification. This result suggests that Patr-B*06:03 has the effect of reducing the VL. (C) When the Patr-B*06:03, B*22:04 and B*39:01 allotypes were tested individually, B*06:03 was again correlated significantly with a lower rate of amplification, whereas B*22:04 and B*39:01 were associated with higher amplification rates. Thus, it is possible that Patr-B*22:04 and Patr-B*39:01 are causing increases in the VL. However, the effect for Patr-B*22:04 is negative (indicative of lower VL) when tested in combination (B). (D) None of the six Patr-B allotypes examined were correlated with either increased or decreased survival of SIVcpz-infected chimpanzees. Applying either the more conservative or less conservative criteria (described in Materials and Methods and S1 Text) gave the same results. Values for Patr-B*39:01 were not reported for the more conservative analysis because only three of the chimpanzees included in the analysis have B*39:01, and by the criteria used in the analysis none of these were categorized as dead. Consequently, the estimate and 95% CI were extraordinarily large and not informative. (TIF) [file pbio.1002144.s018.tif]

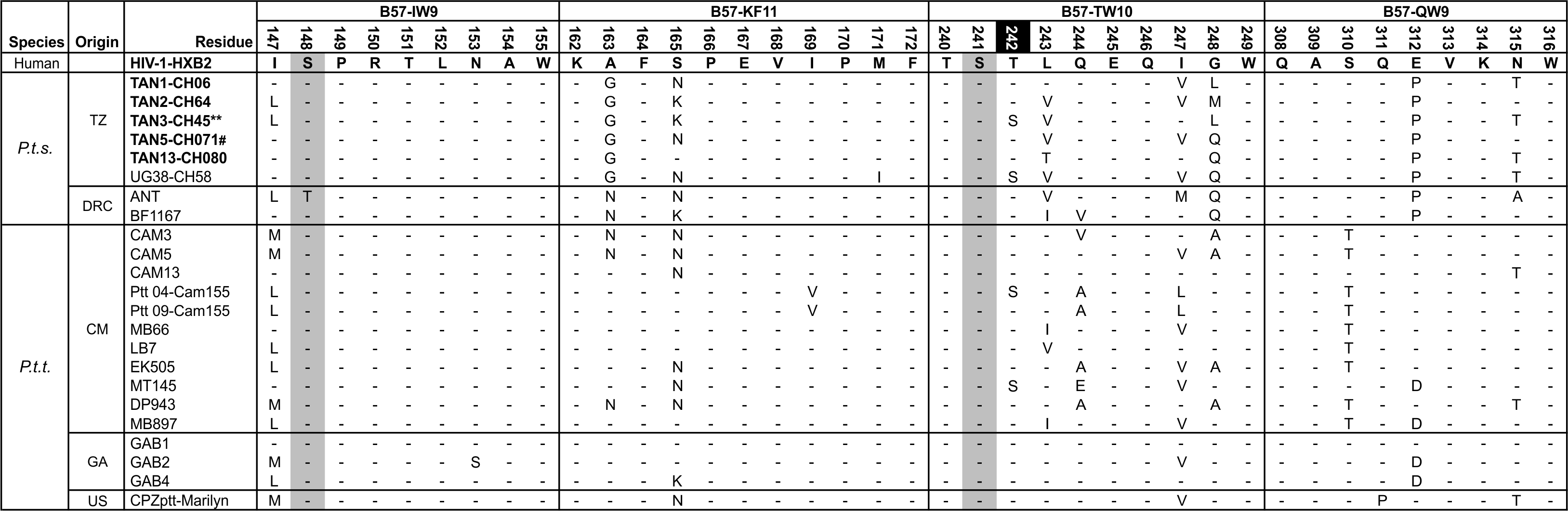

Supplement: S11 Fig — SIVcpz Gag protein sequences were obtained from the HIV sequence database (http://www.hiv.lanl.gov/) and aligned against the four HLA-B*57 restricted HIV-1 Gag epitopes. “P.t.s.” identifies Gag sequences from P. t. schweinfurthii, while “P.t.t.” identifies sequences from P. t. troglodytes. Two epitopes predominantly have serine (S) at the P2 peptide anchor position (in gray). Position 242 (in black) is the site of the T242N HIV-1 viral escape mutation that negatively impacts viral replication potential and frequently occurs in individuals with HLA-B*57 [58,90]. SIVcpz Gag sequences present in the Gombe population are in bold and have an associated, chimpanzee-specific CH identification number (which corresponds to the chimpanzee ID number given in S1 Table). **The individual from which the viral sequence was obtained has Patr-B*06:03 or has Patr-B*22:05. Location of infected animals: Tanzania (TZ), Democratic Republic of the Congo (DRC), Cameroon (CM), Gabon (GA), and the United States (US). (TIF) [file pbio.1002144.s019.tif]

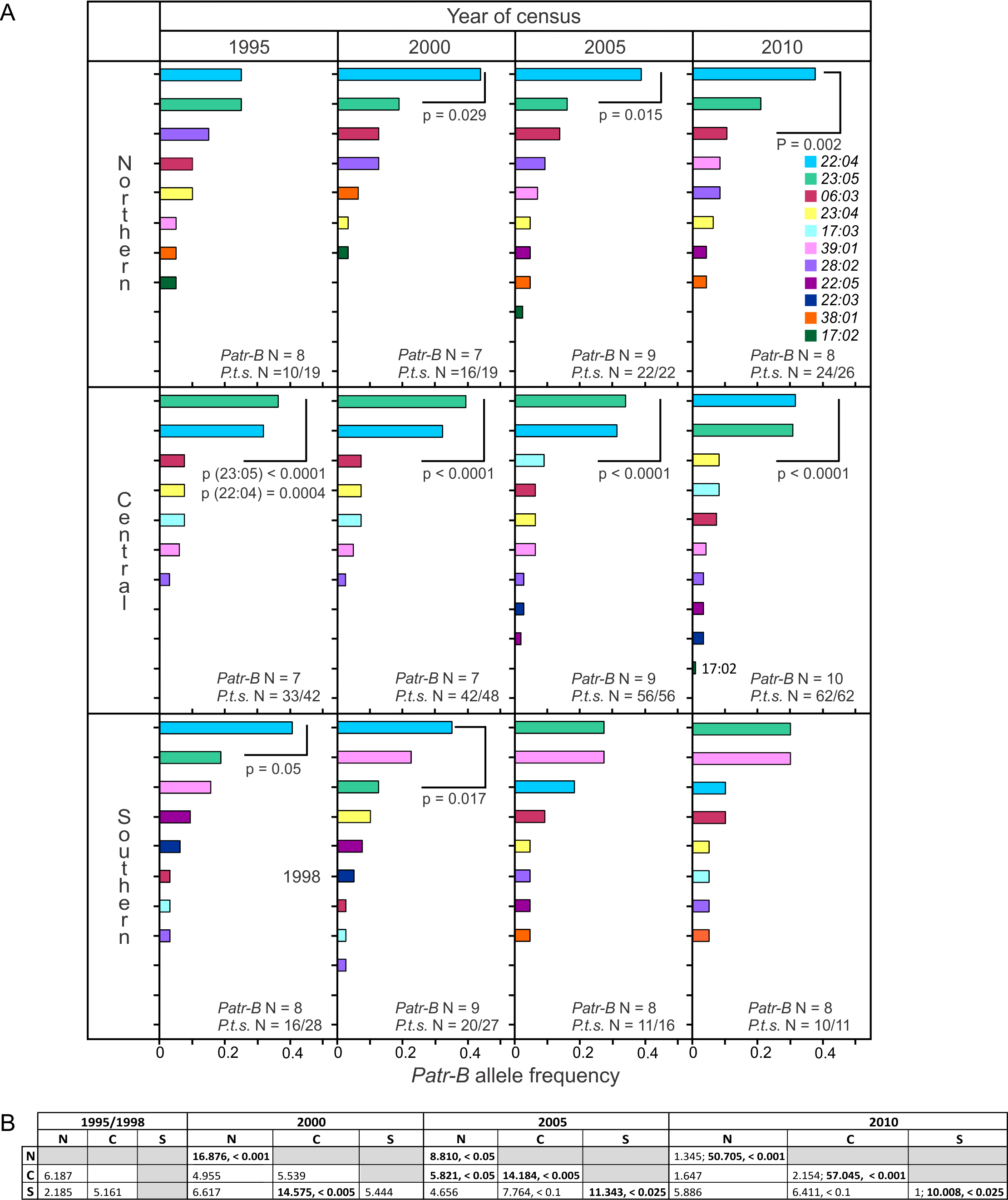

Supplement: S12 Fig — (A) Patr-B N is the number of alleles, while P.t.s N is the number of individuals genotyped for Patr-B out of the total number of individuals alive on the first day of the year. Black brackets indicate significant differences in allele frequencies between the three most frequent alleles within a community at a census time: narrow brackets (south: 1998, north: 2000 and 2005) indicate a difference in the frequencies between the two most frequent alleles; wide brackets in the central community (all years) indicate that the two most frequent alleles are at a significantly higher frequency than the third allele; the bracket between alleles in the southern community in 2000 shows that the highest frequency allele is significantly elevated compared to the third highest frequency allele (Fisher’s Exact tests, p-values given below the brackets (p < 0.0001 applies to both high frequency alleles in the central community)). (B) χ2 test statistics of differences between the total allele frequency distributions within community (compared to the previous year) and between community (within the same year). In this analysis, only the three most common alleles were included individually; all other alleles were combined so as to equalize the number of alleles in the northern (N), central (C), and southern (S) communities. Bold highlights significant results (χ2, significant p-value). Statistics for 2010 first gives the comparison with 2005, and second, the comparison with the earliest time point available for the community (1995 (N and C) or 1998 (S)). Frequencies used in this figure are provided in S4 Data. (TIF) [file pbio.1002144.s020.tif]

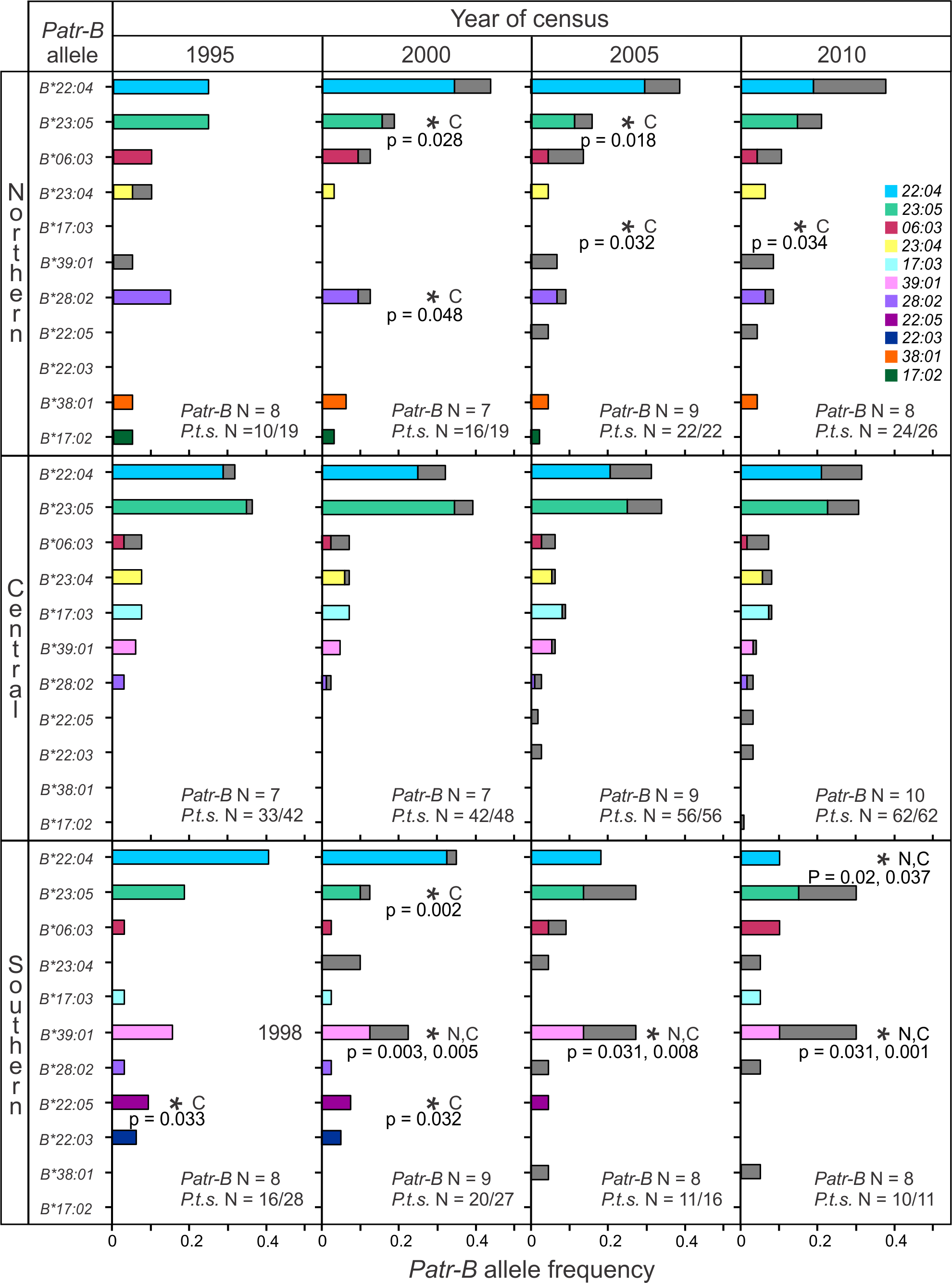

Supplement: S13 Fig — Gray proportions of bars represent the contribution of immigrant females that arrived during the study period and their offspring. Patr-B N represents the number of alleles present within the community. P.t.s N represents the number of individuals that were genotyped for Patr-B out of the total number of individuals alive on the first of the year. Asterisks denote allele frequency differences between communities within the same year (north (N); central (C)) (Fisher’s Exact tests, p-values given below). There were no differences between 1995 (or 1998 for the southern community) and 2010 when comparing within the communities. The data for the northern and southern communities are also presented in Fig 9B and 9C. Frequencies used in this figure are given in S4 Data. (TIF) [file pbio.1002144.s021.tif]

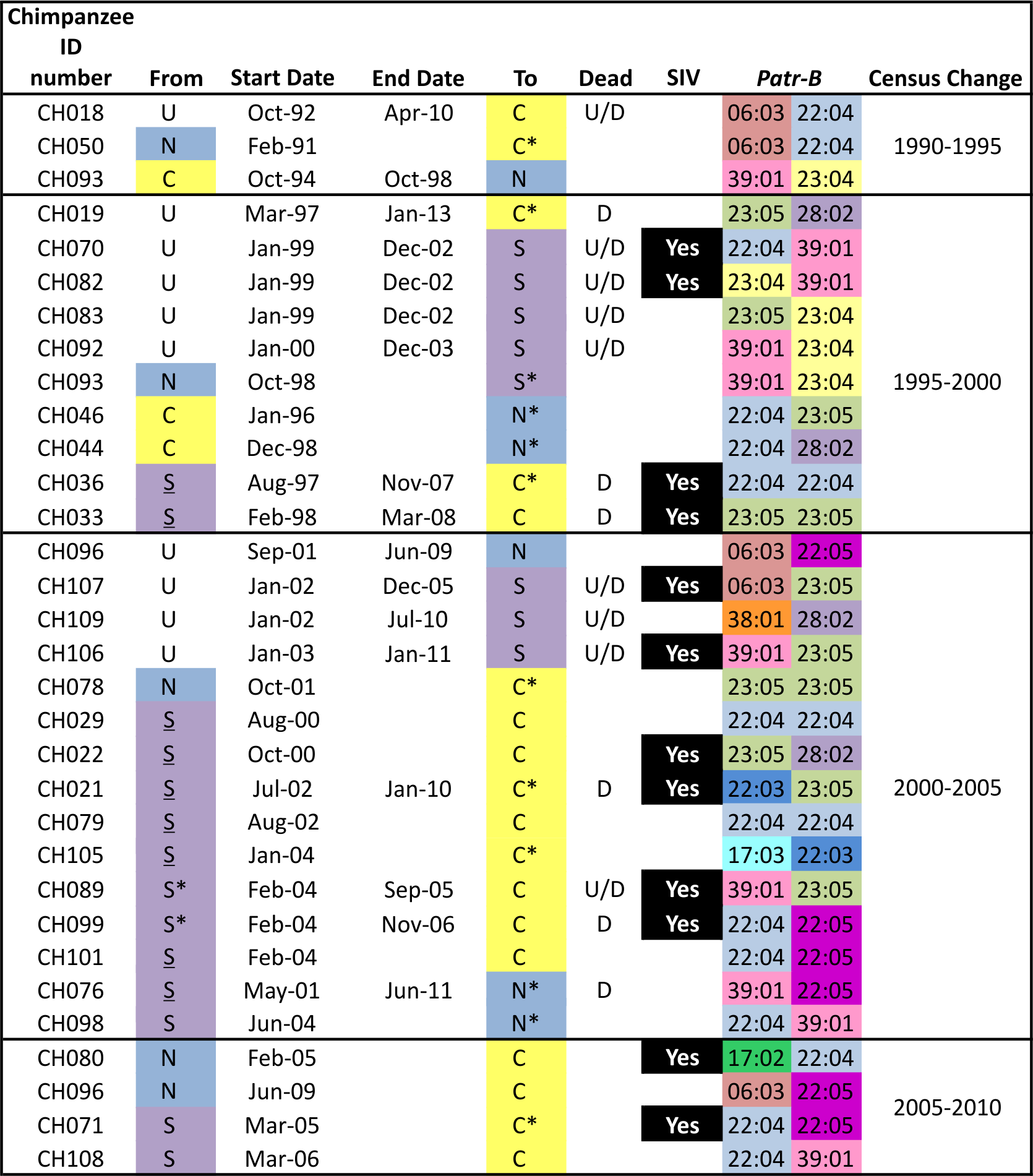

Supplement: S14 Fig — Identification numbers distinguish the females. Females are grouped according to the community “From” which they departed, the community “To” which they went, and then by the date of transfer (“Start Date”, month and year) (unknown (U); north (N, blue); central (C, yellow); south (S, purple). An underlined “S” in the “From” column indicates that the female was inferred to have emigrated from the southern community. Due to the lack of habituation of the southern community, it is possible that some females appearing in the community from unknown origins were actually natal females. Asterisks indicate that the female reproduced within a community. “End Date” lists when the female departed for another community or is null if she still resides there. Two females (CH093 and CH096) had more than one emigration. Females that died (D) or are suspected to have died (U/D) are noted under “Dead.” SIVcpz-infected chimpanzees have “Yes” under “SIV.” The Patr-B genotype for each female is also provided. The females are further grouped according to the Patr-B census years between which their community transfers occurred. (TIF) [file pbio.1002144.s022.tif]

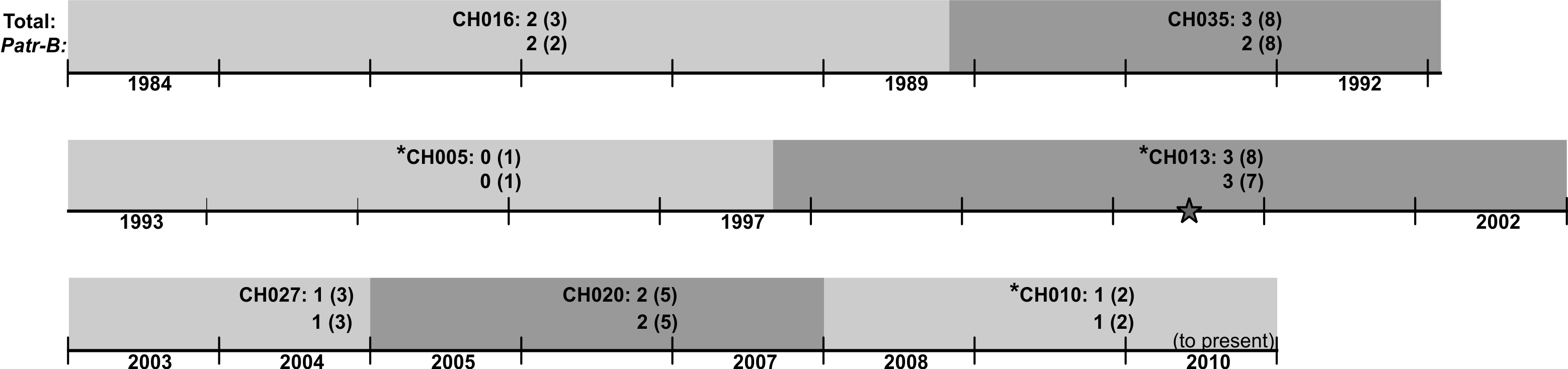

Supplement: S15 Fig — Each timeline increment represents one year. The approximate start and end years of alpha males’ tenures are labeled, and tenure periods alternate between light and dark gray. ID numbers distinguish the alpha males. CH010 was still the alpha male as of the end of 2014. Asterisks identify the F matriline males. The number of offspring sired by each male while occupying the alpha position is given first followed by the total offspring sired (currently known, or lifetime) in parentheses [35,36,79]. The top row of numbers is for all known offspring, while the bottom row is for offspring that were Patr-B genotyped in this study. The star marks the year (2000) in which noninvasive fecal sampling into RNAlater began to obtain SIVcpz vRNA and DNA suitable for sequencing. (TIF) [file pbio.1002144.s023.tif]

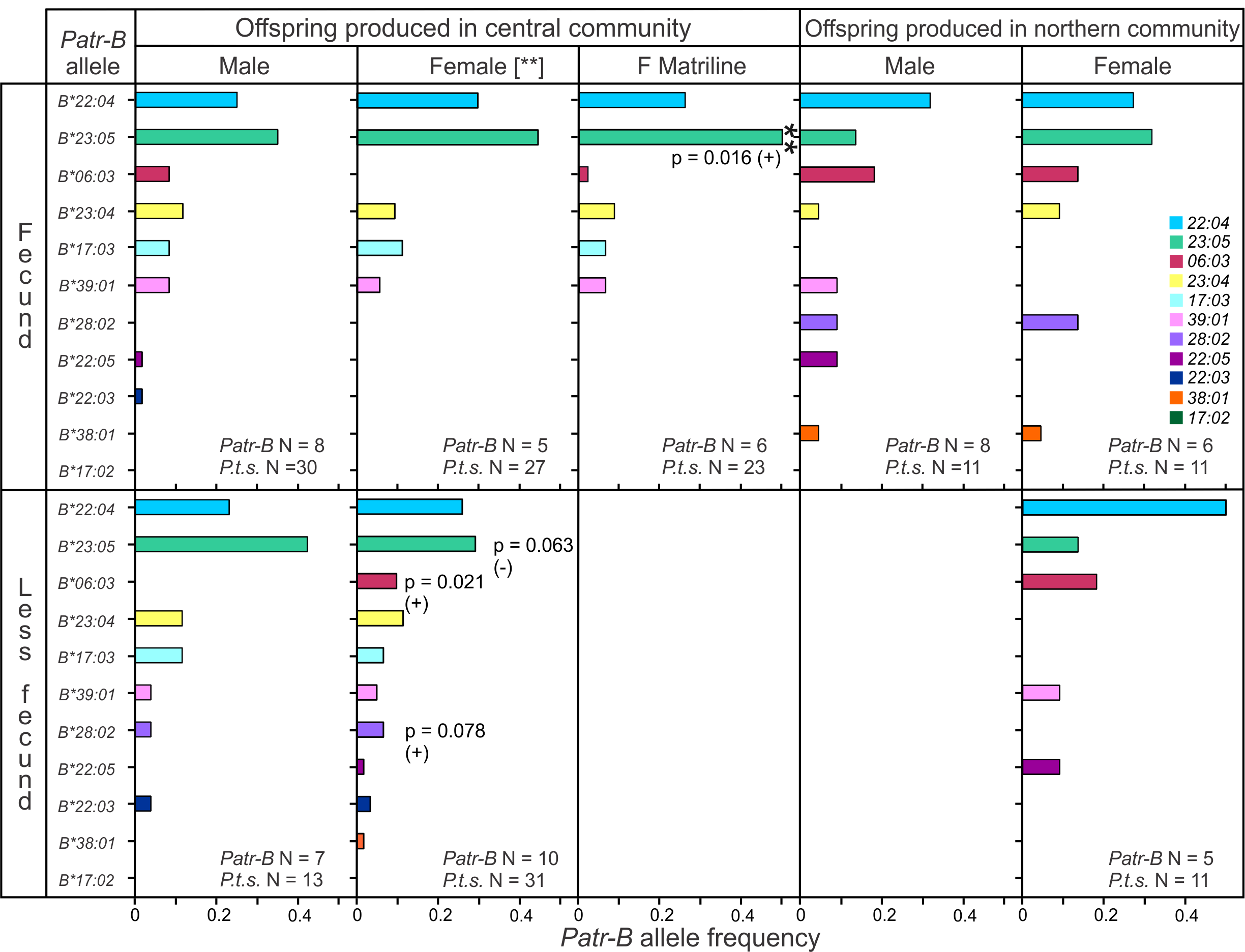

Supplement: S16 Fig — Patr-B N represents the number of alleles present within the offspring produced, while P.t.s N represents the number of offspring produced. Central community offspring with known fathers were sired by seven fecund (alpha) males, and six less fecund males sired the remaining offspring. Central community offspring with known mothers were produced by six fecund females and 23 less fecund females. “F Matriline” represents offspring produced by males and females from three generations of the “F” maternal lineage in the central community. Identified parents of northern community offspring included three fecund (alpha) males, five fecund females, and nine less-fecund females. The frequency distributions of offspring produced by females in the central community were significantly different ([**], χ2 = 8.202, p < 0.05). In this analysis, only the three most common alleles were included individually; all other alleles were combined so as to equalize the number of alleles between the two groups. Differences in individual allele frequencies are noted by giving p-values when they were significantly different between fecund and less fecund distributions, and also ** when they were different than that of the 2010 distribution (Fisher’s Exact tests). A (+) indicates a higher frequency, while (-) indicates a lower frequency. Frequencies used in this figure are provided in S8 Data. (TIF) [file pbio.1002144.s024.tif]
