# Supplementary material for: Signature Patterns of MHC Diversity in Three Gombe Communities of Wild Chimpanzees Reflect Fitness in Reproduction and Immune Defense against SIVcpz
Source: PLoS Biol. 2015 May 28;13(5):e1002144. doi: 10.1371/journal.pbio.1002144 (PMC4447270; doi:10.1371/journal.pbio.1002144)
Supplement: S1 Text — (DOCX) [file pbio.1002144.s027.docx]

**S1 Text**

**Supplemental Materials and Methods**

***Sample collection***

For habituated central and northern chimpanzees collection was made directly after observing defecation. For southern chimpanzees, feces were collected near nest sites. On the occasion of an animal’s death, body parts were collected into RNA*later* (Ambion) during necropsy. Samples obtained from the central community were frozen (-20°C) on the same day of collection, whereas samples from the northern and southern communities were not frozen until they had been transported to the field lab in Gombe National Park (typically within a week). Samples were then shipped by air at ambient temperature from Dar es Salaam, Tanzania to the laboratory of Beatrice Hahn in the United States and subsequently stored frozen at -80°C (total transit time from Gombe National Park to the Hahn laboratory is about one week).

***DNA extraction***

We used the QIAamp DNA Stool Mini Kit (Qiagen), with a modified protocol, to extract DNA from duplicate fecal sample aliquots of 750 µl of fecal-RNA*later* mixture (1.5 ml total volume per sample). Each sample aliquot was processed separately until the final elution, and all centrifugations were at 20,000 g (~14,000 rpm). Aliquots were incubated in 1.25 ml of ASL lysis buffer (vortexed well for 1 minute) for 20-30 minutes at 70°C. After incubation, aliquots were vortexed again (1 minute) and then centrifuged for one minute. Supernatants were then transferred to a fresh tube and vortexed 30-60 seconds with half of a QIAamp InhibitEX tablet. After a second centrifugation (3 minutes), 900 µl of supernatant was transferred to a new tube containing 25 µl of proteinase K, and 900 µl of buffer AL was then added to each tube. After vortexing briefly, aliquots were again incubated at 70°C for at least 10 minutes (typically 30-45 minutes). Each sample aliquot was then split between two tubes (~900 ul each), and 450 µl of EtOH was added to each tube and briefly vortexed. Each aliquot (split between the two tubes) was passed through a single QIAamp DNA binding column in successive additions of no more than 650 µl, with a centrifugation (1 minute) between volume applications. Each aliquot was then washed with 500 µl of AW1 and then AW2 buffer, centrifuging samples (1 minute) after each wash. After a dry centrifugation (1 minute), 130 µl of buffer AE was added to one sample aliquot column and 50 µl of AE was added to the second sample aliquot column. Columns were incubated for 20 minutes, and then the 130 µl column was centrifuged for two minutes. The eluate was then transferred to the second column (already containing 50 µl AE), and incubated again for 20 minutes. A final two minute centrifugation resulted in a single DNA extraction for each, individual fecal sample in ~180 µl total volume.

***PCR amplification and sequencing of Patr-B***

Primers were designed from conserved intronic sequences flanking exons 2 and 3. Exon 2 primers amplified a 425 bp fragment (B2-425FOR-5’-ATGGCCTCTGCCGGGAGGAG-3’ and B2-425REV-5’-CGTSGGGGWTGGGGAGTCGT-3’). Exon 3 primers (B3-411FOR-5’-GGCCAAAATCCCCGCGGGTT-3’ and B3-411REV-5’-GTGGGAGGCCATCCCSGGC-3’) amplified a 411 bp fragment. The B2-425 primer pair does not amplify *Patr-B*17* sequences, probably because of a 3 bp insertion in the reverse primer sequence in intron 2, as observed in *Patr-B*17:01*. Consequently, a separate *Patr-B*17*-specific reverse primer (B2-429REV-5’-GGGTTGGGGAGGGGTCGTGA-3’) was used with the B2-425FOR primer to give a 429 bp amplicon of *Patr-B*17*-specific exon 2 sequences. This specific reaction for exon 2 was performed for samples for which a *Patr-B*17* exon 3 had been amplified. It was used for individuals who were homozygous in exon 2 with the B2-425 primer pair (unless the individual had parents both known to lack *Patr-B*17*). Similarly, primers for exon 3 were designed to specifically amplify the *Patr-B*17* sequence of exon 3 of the chimpanzee, CH080 (Table S1), because *Patr-B*17:02* was detected in exon 2 with the *Patr-B*17*-specific PCR, but the standard exon 3 (B3-411) primer pair did not amplify a *Patr-B*17* sequence. The primers therefore encompassed single nucleotide polymorphisms (SNPs) specific to intron sequence of *Patr-B*17:01* and amplified a 446 bp amplicon (B3-446FOR-5’-CAGAGAGCCCCAGGCGAC-3’ and B3-446REV-5’-ATCCCGGGCGATCTATAGGAGA-3’). Primer pairs were first validated and optimized on matched DNA extracts from feces and peripheral blood mononuclear cells (PBMCs) from at least three captive chimpanzees housed at Yerkes Regional Primate Research Center (Atlanta, Georgia). PBMCs were isolated from blood samples by Ficoll gradient separation. DNA was subsequently extracted from the cells using the QIAamp DNA Blood kit (Qiagen).

In characterizing the Gombe chimpanzees, PCR amplifications were performed on 160 ng of total DNA in 25 ul reactions containing: 0.25 mM each dNTP (Promega), 0.2 µM of each primer, 1 unit of Hot Star Taq Plus polymerase (Qiagen) and 1x Hot Star Taq Plus buffer. The exon 3 reactions with the B3-411 and B3-446 primer pairs also contained 1 M Betaine (Sigma). The reactions underwent initial denaturation for 5 min at 95°C followed by 45 cycles of amplification (95°C for 30 s, annealing for 45 s, and 72°C for 40s), with a final extension of 10 min at 72°C. Annealing temperatures were 65°C for the B2-425 and B2-429 reactions, 67°C for the B3-411 reaction, and 59°C for the B3-446 reaction. To identify SNPs, PCR products were first sequenced directly with at least one of the amplification primers. Whenever a new allele was detected, or if results were unclear, the PCR products were cloned using the TOPA TA cloning kit (Invitrogen) and then sequenced using the M13F vector site. At least 12 clones were sequenced for each allele.

***BPRC population allele frequencies***

The colony of 32 founder *Pan troglodytes verus* chimpanzees at the Biomedical Primate Research Center (BPRC) in the Netherlands was used for comparison to the Gombe chimpanzee *P. t. schweinfurthii* population [1]. Seven BPRC chimpanzees were typed for only one of their two *Patr-B* alleles. Their 7 unknown alleles were still included in the total allele number for the population (64 total alleles, rather than 57 identified) in order to calculate exact allele frequencies for the other, known alleles.

***SIVcpz mortality analyses***

Per Keele et al. 2009 [2], analyses were based on 1 year periods of individual observation, during which life events such as SIV infection (yes/no) and death (yes/no) were noted whether to have occurred within that period. Given this data, we then used a binomial generalized linear model, or logistic regression, to model SIVcpz infected chimpanzee death with allele presence, age, and sex as covariates in SAS v9.3 (proc GENMOD). We also used the complementary log-log link, which is appropriate to such discrete event (interval) data, as opposed to survival based on continuous time. We used this model to conduct conservative and less conservative analyses according to individuals*’* SIV status and dates of death. Thus only individuals positive for specific antibody and viral RNA were considered infected in the conservative analysis, whereas individuals positive for antibody and lacking viral RNA were also considered infected in the less conservative analysis. Absent individuals, for whom death was uncertain, were considered alive in the conservative analysis but dead in the less conservative analysis.

**References**

1. de Groot NG, Heijmans CMC, Zoet YM, de Ru AH, Verreck FA, et al. (2010) AIDS-protective HLA-B*27/B*57 and chimpanzee MHC class I molecules target analogous conserved areas of HIV-1/SIVcpz. Proc Natl Acad Sci 107: 15175-15180.

2. Keele BF, Jones JH, Terio KA, Estes JD, Rudicell RS, et al. (2009) Increased mortality and AIDS-like immunopathology in wild chimpanzees infected with SIVcpz. Nature 460: 515-519.
